# Photo-thermal coupling to enhance CO_2_ hydrogenation toward CH_4_ over Ru/MnO/Mn_3_O_4_

**DOI:** 10.1038/s41467-024-45389-7

**Published:** 2024-02-06

**Authors:** Jianxin Zhai, Zhanghui Xia, Baowen Zhou, Haihong Wu, Teng Xue, Xiao Chen, Jiapeng Jiao, Shuaiqiang Jia, Mingyuan He, Buxing Han

**Affiliations:** 1 Shanghai Key Laboratory of Green Chemistry and Chemical Processes, State Key Laboratory of Petroleum Molecular & Process Engineering, School of Chemistry and Molecular Engineering, East China Normal University, Shanghai, 200062 China; 2 Institute of Eco-Chongming, Shanghai, 202162 China; 3 Key Laboratory for Power Machinery and Engineering of Ministry of Education, Research Center for Renewable Synthetic Fuel, School of Mechanical Engineering, Shanghai Jiao Tong University, Shanghai, 200240 China; 4 Beijing National Laboratory for Molecular Sciences, CAS Key Laboratory of Colloid and Interface and Thermodynamics, CAS Research/Education Center for Excellence in Molecular Sciences, Institute of Chemistry, Chinese Academy of Sciences, Beijing, 100190 China

**Keywords:** Sustainability, Heterogeneous catalysis

## Abstract

Upcycling of CO_2_ into fuels by virtually unlimited solar energy provides an ultimate solution for addressing the substantial challenges of energy crisis and climate change. In this work, we report an efficient nanostructured Ru/MnO_x_ catalyst composed of well-defined Ru/MnO/Mn_3_O_4_ for photo-thermal catalytic CO_2_ hydrogenation to CH_4_, which is the result of a combination of external heating and irradiation. Remarkably, under relatively mild conditions of 200 °C, a considerable CH_4_ production rate of 166.7 mmol g^−1^ h^−1^ was achieved with a superior selectivity of 99.5% at CO_2_ conversion of 66.8%. The correlative spectroscopic and theoretical investigations suggest that the yield of CH_4_ is enhanced by coordinating photon energy with thermal energy to reduce the activation energy of reaction and promote formation of key intermediate COOH* species over the catalyst. This work opens up a new strategy for CO_2_ hydrogenation toward CH_4_.

## Abstract (web summary)

Reduction of CO_2_ with solar energy is an attractive route to new fuel sources. Here a nanostructured Ru/MnO_x_ photo-thermal catalyst composed of well-defined Ru/MnO/Mn_3_O_4_ is reported for CO_2_ hydrogenation with high selectivity and a CH_4_ production rate at a mild temperature of 200 °C.

## Introduction

Upcycling of CO_2_ into fuels with the use of green hydrogen presents a promising route for addressing the challenges of energy crisis and climate change^1–3^. Among a variety of products from CO_2_ hydrogenation, CH_4_ is regarded as an ideal energy vector owing to its merits of high energy density, widely available infrastructure of storage, transportation, and utilization^4,5^. To date, a broad range of catalytic systems have been developed for CO_2_ hydrogenation toward CH_4_^6^. However, because of the inert nature of CO_2_ and complex reaction network, efficient production of CH_4_ from CO_2_ hydrogenation is challenging, suffering from unsatisfactory activity, harsh reaction condition and extensive thermal input^7–9^. It is imperative to explore new and green methods for the conversion of CO_2_ toward CH_4_.

Photo-thermal-catalysis presents a synergistic configuration for mediating chemical reactions by simultaneously taking advantage of charge carriers and thermal energy^10,11^. Thus far, there is a growing number of researches on photo-thermal catalytic CO_2_ hydrogenation toward CH_4_ and remarkable progress has been made^12,13^. For example, Liu et al. reported that Co_7_Cu_1_Mn_1_O_*x*_ (200) was active for CH_4_ synthesis from CO_2_ hydrogenation with an production rate of 14.5 mmol g^−1^ h^−1^ and a selectivity of 85.3% at 200 °C^14^. Zou’s group demonstrated that Ru@Ni_2_V_2_O_7_ catalyst exhibited CH_4_ production rate of 114.9 mmol g^−1^ h^−1^ and 99.3% selectivity at 350 °C^15^. Overall, the performance of the catalytic systems is still far away from practical applications and the reaction mechanism remains largely unknown^16^. It is very desirable to explore a strategy for mediating CO_2_ hydrogenation toward CH_4_ with high efficiency and selectivity^17^.

Among a broad range of CO_2_ hydrogenation catalysts, Ru-based catalysts exhibit great potential in CO_2_ hydrogenation toward CH_4_ because of their unique catalytic properties^18^. Apart from metal centers, the support also plays a critical role in CO_2_ hydrogenation by influencing the geometric and electronic properties of active sites. Particularly, MnO_x_ is considered a promising support for hydrogenation reactions due to some obvious advantages^19^. It is worth of noting that the multiple valences and reducible effect of MnO_x_ confers flexible mediation capability on the catalysts^20^. The integration of Ru species with MnO_x_ is thus highly promising for efficient CO_2_ hydrogenation toward CH_4_.

In this work, a nanostructured Ru/MnO_x_ photo-thermal catalyst composed of well-defined Ru/MnO/Mn_3_O_4_ at reaction temperature was designed and prepared for CO_2_ hydrogenation toward CH_4_. A prominent CO_2_ conversion of 66.8% was achieved with a superior selectivity of 99.5% and a CH_4_ production rate of 166.7 mmol g^−1^ h^−1^ at relatively mild temperature of 200 °C (normalized by the amount of catalyst (~15 mg)), which is the result of a combination of external heating and irradiation. The correlative spectroscopic characterizations and theoretical calculations revealed that the structural evolution of Ru/MnO_x_ into well-defined Ru/MnO/Mn_3_O_4_ was facilitated by Ru-mediated H-spillover in MnO_x_ and the activity was enhanced by the synergistic effects of photon energy and thermal energy via reducing the activation energy of reaction and accelerating the key intermediate of COOH* species formation over the catalyst.

## Results

### Fabrication and characterization of Ru/MnO_x_

**Fig. 1:**
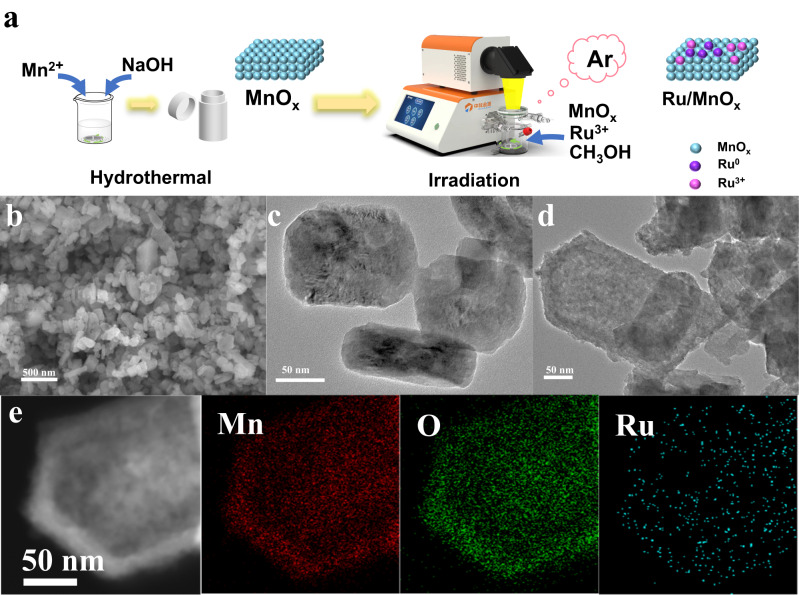
Structural characterization of the catalysts. **a** Schematic illustration of the synthesis of Ru/MnO_x_; **b** SEM image of MnO_x_; **c**, **d** TEM image of MnO_x_ and Ru/MnO_x_; **e** STEM image and elemental mapping of Ru/MnO_x_.

The synthesis process of Ru/MnO_x_ was schematically shown in Fig. 1a. Typically, MnO_x_ nanoparticles were first prepared via a straightforward hydrothermal method. Ru sites were then anchored onto MnO_x_ by photo-deposition under argon atmosphere^21^. The content of Ru in Ru/MnO_x_ was measured by inductively coupled plasma optical emission spectrometry (ICP-OES) (Supplementary Table 1). If not specifically noted, the content of Ru in Ru/MnO_x_ was referred to be 7.3 wt%. The morphologies and structures of the synthesized materials were characterized by scanning electron microscopy (SEM) and transmission electron microscopy (TEM). As shown in Figs. 1b, c, MnO_*x*_ displayed variable morphologies of hexagonal, octahedral, and square schistose crystals. The morphology of MnO_x_ did not change considerably after the addition of Ru species and the average size of the deposited Ru nanoclusters is about 1.07 ± 0.26 nm (Fig. 1d and Supplementary Figs. 1, 2). The energy dispersive spectroscopy elemental mapping in Fig. 1e exhibited the even distribution of Mn, O, and Ru, which is indicative of the successful synthesis of Ru/MnO_x_.

The Rietveld refinement of X-ray powder diffraction (XRD) results in Supplementary Fig. 3 and Supplementary Table 2 indicated that the MnO_x_ nanoparticles were mainly composed of Mn_3_O_4_ (JCPDS No. 80-0382), MnO_2_ (JCPDS 72–1806) and MnOOH (JCPDS No. 18-0804)^22–24^. Meanwhile, the XRD pattern of Ru/MnO_x_ showed that the content of MnO_2_ phase decreased slightly, indicating that the process of photo-deposition of Ru had a slight reduction effect on MnO_2_. The structure of the samples was further characterized by Fourier transform infrared spectroscopy (FT-IR) spectroscopy (Supplementary Fig. 4). The peaks at 513 and 621 cm^−1^ were attributed to the distortion vibration of Mn-O in octahedral sites and Mn-O stretching modes in tetrahedral sites, respectively^25^. Besides, the typical peaks at 947 and 1074 cm^−1^ were attributed to the vibration of hydroxyl in MnOOH^26^. Meanwhile, Raman spectrometer was employed to study the metal-support interaction between Ru and MnO_x_. As illustrated in Supplementary Fig. 5, compared with the pristine MnO_x_, the introduction of Ru species led to a blue shift of ~7 wavenumbers, and the main peak at 637 cm^−1^ is assigned to *A*_1g_ mode of crystalline Mn_3_O_4_, validating the strong metal–support interaction between Ru and MnO_x_^27^. Moreover, as characterized by CO_2_ adsorption isotherm and N_2_ adsorption–desorption isotherms, the addition of Ru species enhanced the CO_2_ adsorption capacity; and specific surface area of the catalyst was enlarged accordingly (Supplementary Figs. 6, 7). Such improvements are beneficial for the interaction between reactants and mass transfer, thus facilitating CO_2_ methanation. In addition, X-ray photoelectron spectroscopy (XPS) was examined to gain more insight into surface chemical state and electronic structure of the catalysts (Supplementary Fig. 8). For Ru/MnO_x_, Ru^0^ is identified by the peaks observed at ca. 462.7 and 484.8 eV, implying the reduction of Ru species under light irradiation^28^. Additionally, XPS analysis of the Mn species in all tested samples can be deconvolved into Mn^4+^, Mn^3+^, and Mn^2+^^23,27^. Apparently, multiple valences of MnO_x_ indicate the reducible nature of as-prepared catalyst.

### Photo-thermal catalytic CO_2_ hydrogenation

**Fig. 2:**
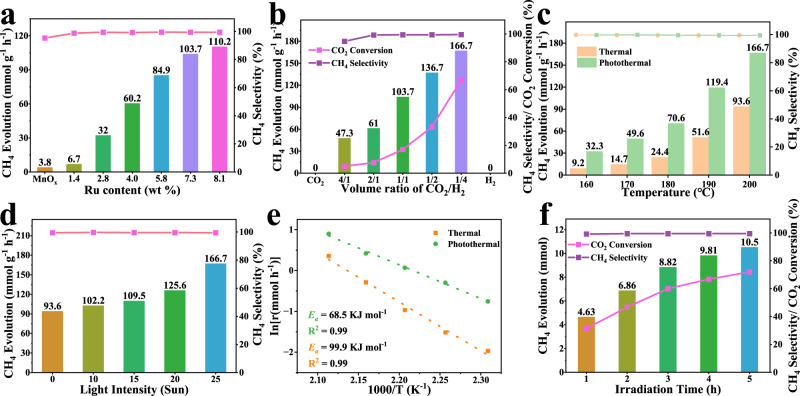
Photo-thermal-catalytic performance. **a** Influence of the Ru content on CH_4_ evolution rate over Ru/MnO_x_; **b** Influence of CO_2_/H_2_ volume ratio in the feedstock on CH_4_ evolution rate over Ru/MnO_x_; **c** Temperature-dependent CH_4_ generation rate over Ru/MnO_x_ under photo-thermal and thermal conditions; **d** Influence of light intensity on CH_4_ evolution rate over Ru/MnO_x_; **e** Corresponding Arrhenius plot with activation energies noted under photo-thermal/thermal conditions over the Ru/MnO_*x*_ catalysts. **f** CH_4_ evolution as a function of reaction time over Ru/MnO_*x*_. Reaction conditions: 15 mg of catalyst, full-arc 300 W UV-xenon lamp, 2.5 W cm^−2^, 200 °C, irradiation time 4 h, initial pressure 1 MPa (H_2_/CO_2_ = 1/1) for Fig. 2a or initial pressure 1 MPa (H_2_/CO_2_ = 4/1) for Fig. 2c–f.

The catalytic performance of Ru/MnO_x_ was evaluated at 200 °C in the batch reactor setup by feeding CO_2_/H_2_ mixed gas (the desired temperature was achieved by a combination of external heating and irradiation from the Xe lamp) and CH_4_ was identified as the dominant product, with no liquid products produced (Supplementary Figs. 9, 10). As shown in Fig. 2a, MnO_x_ was hardly active for CO_2_ hydrogenation toward CH_4_. In contrast, after the addition of Ru species into MnO_x_, considerable activity for CH_4_ formation was achieved, indicating that Ru species could serve as effective active site for the reaction. Notably, the catalytic activity of Ru/MnO_x_ gradually increased with an increasing amount of Ru. At a Ru content of 7.3 wt%, the catalyst displayed a decent CH_4_ activity of 103.7 mmol g^−1^ h^−1^. The CH_4_ activity was increased by further increasing the loading content of Ru, but the trend slowed down. The effect of CO_2_/H_2_ ratios on the yield of CH_4_ was then examined. It was observed that the yield of CH_4_ monotonically increased with the H_2_ proportion in the H_2_/CO_2_ mixture (Fig. 2b). A distinct CH_4_ production rate of 166.7 mmol g^−1^ h^−1^ was obtained for Ru/MnO_x_ at a relatively high H_2_/CO_2_ ratio of 4/1, highlighting the importance of adequate H_2_ supply during the reaction to enhance its activity. Furthermore, as shown in Supplementary Fig. 11, we studied the influence of the total pressure on the reaction at high H_2_/CO_2_ ratio (4/1). The activity was enhanced markedly with the elevating total pressure, but became slowly when the pressure exceeded 1 MPa.

As shown in Supplementary Fig. 12, the control experiment showed that no carbonaceous products were detected without catalyst or reactant gas, confirming that CH_4_ was catalytically produced from CO_2_ hydrogenation. Furthermore, when the photon energy was coupled with external thermal energy, the evolution rate of CH_4_ was substantially enhanced compared to that achieved by thermo-catalysis in the temperature range examined (Fig. 2c). In addition, when the reaction temperature was kept at 200 °C by an external temperature-controlling system, the CH_4_ evolution rate of Ru/MnO_x_ could be further enhanced by increasing the light intensity, reaching 166.7 mmol g^−1^ h^−1^ at 2.5 W cm^−2^ (Fig. 2d). These results provide solid support that the introduction of photon energy can significantly enhance CO_2_ hydrogenation reaction. Meanwhile, the activation energies of Ru/MnO_x_ under thermal and photo-thermal conditions were estimated to be 99.9 and 68.5 kJ mol^−1^, respectively (Fig. 2e). The decreased activation energy and the corresponding non-parallel plots were indicative of a synergy between photon energy and thermal energy, altering the catalytic mechanism when photons were involved. Moreover, the conversion of CO_2_ toward CH_4_ as a function of reaction time is shown in Fig. 2f. It was found that the yield of CH_4_ continuously increased with the reaction time, suggesting the continuous generation of CH_4_ from CO_2_ hydrogenation over Ru/MnO_x_. Impressively, with a reaction time of 4 h at 200 °C, a considerable CO_2_ conversion of 66.8% was achieved with a high selectivity of 99.5% with an appreciable CH_4_ production rate of 166.7 mmol g^−1^ h^−1^. Furthermore, the photothermal catalytic performance of the Ru/MnO_x_ catalyst was also assessed in a fixed-bed reactor. As shown in Supplementary Figs. 13, 14, at a gas hourly space velocity (GHSV) of 20,000 mL g^−1^ h^−1^, the catalytic activity of Ru/MnO_x_ gradually increased with an increasing temperature, and its activities under photothermal conditions were higher than those under thermal conditions, which further proves that the involved photons were prone to promote the formation of CH_4_ in the fixed-bed reactor. Meanwhile, under the conditions of 200 °C and 2.5 W cm^−2^ irradiation, the catalytic activity of Ru/MnO_x_ remained acceptably stable after 20 h at a high GHSV of 40,000 mL g^−1^ h^−1^ (Supplementary Fig. 15). A CO_2_ conversion of 29.5% was achieved with an excellent selectivity of 99.5% and a high space-time yield (STY) of $95.8\,{{{\rm{mmol}}}_{{CH}_{4}}}$ g^−1^ h^−1^. The decrease in activity was probably caused by catalyst agglomeration and carbon deposition as confirmed by the TEM and Thermogravimetric-mass spectrometric (TG-MS) of the catalyst after the reaction (Supplementary Figs. 16, 17). In addition, as characterized by XPS, for Ru/MnO_x_, after the reaction, the composition of Mn^4+^ disappeared, and the peak appearing at 647 eV is the satellite peak of Mn^2+^, demonstrating that the multiphase MnO_x_ support underwent partial reduction^29^. Ru species were completely converted into Ru^0^, indicating that the in-situ generated Ru can act as a metal active center to enhance the dissociation of H_2_ and spillover of hydrogen to the MnO_x_ support (Supplementary Fig. 18). Therefore, the actual composition of the catalyst during the reaction was altered, and the observations above suggested the H-spillover effects in Ru/MnO_x_, which will be discussed further in the following.

### Origin of the superior activity over Ru/MnO_x_

**Fig. 3:**
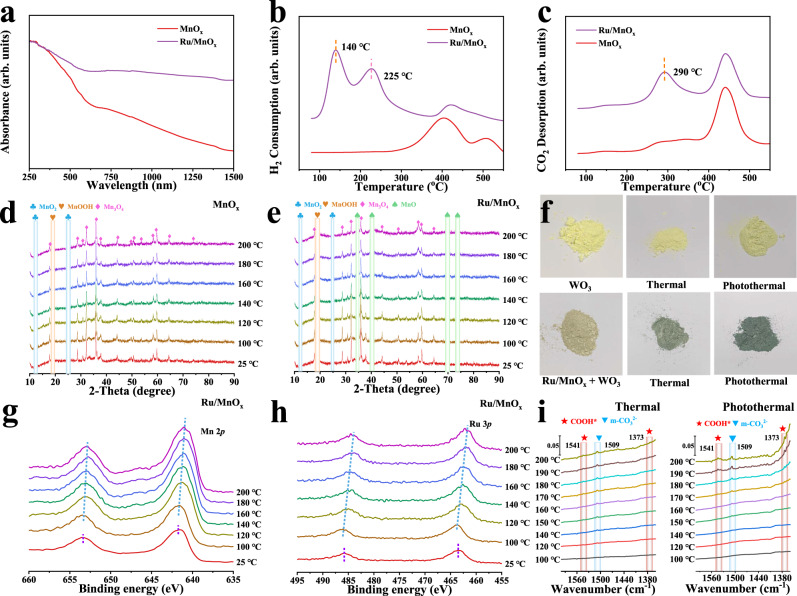
The mechanism analysis. **a** UV–Vis–IR absorption spectra of MnO_x_ and Ru/MnO_x_; **b** H_2_−TPR and **c** CO_2_-TPD characterization for MnO_x_ and Ru/MnO_x_; **d**, **e** The variable-temperature XRD patterns of MnO_x_ and Ru/MnO_x_ recorded under 20% CO_2_/H_2_ atmosphere at different temperatures; **f** Photographs of WO_3_ and the mixture of Ru/MnO_x_ and WO_3_ samples after treatment with H_2_ at 80 °C with a light intensity of 0.3 W cm^−2^ for 20 min; **g**, **h** XPS spectra of Ru/MnO_x_ in 20% CO_2_/H_2_ atmosphere under variable temperature; **i** Spectra of FT-IR study of Ru/MnO_x_ at different conditions.

The optical and electronic properties of the catalyst play a vital role in photo-thermal-catalytic activity. Thereby, they were characterized by various spectroscopy techniques. Firstly, the UV–Vis–NIR diffuse reflectance spectra of different samples were measured to study the light absorption capacity. As illustrated in Fig. 3a, MnO_x_, as an excellent semiconducting support, exhibits suitable light absorption in the UV–Vis region. The light absorption of MnO_x_ was further improved by the addition of Ru species, having stronger and broader light absorption from UV to NIR wavelength. Meanwhile, due to the broadening of the wavelength range of light absorption, a strong photothermal effect was expected^30,31^. As shown in Supplementary Fig. 19, under 2.5 W cm^−2^ illumination, the measured average temperature of Ru/MnO_x_ reached 137.9 °C, higher than that of MnO_x_ (115.4 °C), indicating that both Ru and MnO_x_ contributed to the photothermal effect. In addition, time-resolved photoluminescence spectroscopy showed that the charge carriers lifetime of bare MnO_x_ (*τ*_1_ = 1.05 ns, *τ*_2_ = 13.51 ns) was obviously prolonged by the incorporation of Ru species (*τ*_1_ = 1.33 ns, *τ*_2_ = 17.14 ns for Ru/MnO_x_) in Supplementary Fig. 20^32^. The behavior of charge carriers was further investigated by transient photocurrent spectra, and the results are plotted in Supplementary Fig. 21. Under light irradiation, Ru/MnO_x_ exhibits a significantly higher photocurrent intensity than MnO_x_^33^. The results above show that both optical and electronic properties of MnO_x_ were enhanced by the introduction of Ru species. Moreover, the flat band potentials of MnO_x_ were investigated by Mott-Schottky plots. As shown in Supplementary Fig. 22a, MnO_x_ is confirmed as a p-type semiconductor due to the negative slope. Meanwhile the valence band edge potential was evaluated to be c.a. 0.29 (0.49 eV vs. NHE, *E*_NHE_ = *E*_Ag/AgCl_ + 0.197), while the flat-band potential is 0.1–0.3 eV lower than the valence band potential in the p-type semiconductor^34^. In addition, the band gap can be estimated to be 1.26 eV for MnO_x_ (Supplementary Fig. 22b). Consequently, as shown in Supplementary Fig. 23, considering that the work function of Ru is 4.71 eV, the photo-excited electrons can facilely transfer from MnO_x_ to Ru sites under light irradiation^35^. Together with the unique catalytic properties, the assembled photo-thermal catalyst of Ru/MnO_x_ was therefore highly efficient and selective for catalyzing CO_2_ hydrogenation toward CH_4_.

The characterizations of H_2_ temperature programmed reduction (H_2_-TPR) and CO_2_ temperature programmed desorption (CO_2_-TPD) were performed to figure out the important role of Ru and MnO_x_ in CO_2_ methanation. Compared to pristine MnO_x_, Ru/MnO_x_ exhibited remarkably lowered H_2_ reduction temperature, which was as low as 140 °C (Fig. 3b), suggesting the superior H_2_ activation capacity of Ru species. The temperature of H_2_ activation over Ru/MnO_x_ was even much lower than that for CO_2_ hydrogenation (200 °C). Hence, CO_2_ hydrogenation could benefit from low-temperature H_2_ activation by Ru species under experimental conditions. Moreover, upon the introduction of Ru species, the reduction temperature peaks of MnO_x_ were observed to emerge at low temperature range of 180–250 °C. Herein, the partial reduction of the support MnO_x_ was probably associated with the hydrogen dissociation on Ru and its subsequent spillover to MnO_x_^36^. The CO_2_ adsorption properties were then evaluated by CO_2_-TPD. As shown in Fig. 3c, compared to pristine MnO_x_, Ru/MnO_x_ showed remarkable desorption peaks between 250 and 350 °C, indicating that Ru species obviously enhanced the adsorption of CO_2_^37^. Therefore, the dissociated H can further modulate the hydrogenation activity by directly reacting with CO_2_ adsorbed on Ru sites or MnO_x_ support, thereby exerting a positive impact on the methanation.

To further explore the Ru-mediated H-spillover effect on the structural composition of Ru/MnO_x_, qualitative analysis of the crystal phases of Ru/MnO_x_ during the CO_2_ methanation process was conducted by variable temperature XRD measurements, with a focus on revealing the structural evolution of MnO_x_ at different stages. As illustrated in Fig. 3d, e, all the tested samples are primarily composed of Mn_3_O_4_, MnO_2_, and MnOOH. For MnO_x_, MnO_2_ phase can be reduced when the temperature reaches 100 °C. Meanwhile, the transformation of MnOOH to Mn_3_O_4_ phase is observed between 100 and 200 °C. In contrast, the Mn_3_O_4_ phase remains stable at least 4 h at 200 °C (Supplementary Fig. 24). Interestingly, upon the introduction of Ru species, the variable temperature XRD patterns of Ru/MnO_x_ showed notable changes. The terminal transforming temperature of MnOOH phase markedly decreases from 200 °C to 140 °C. Then, MnO (JCPDS No. 80-0382) with diffraction peaks at 34.7° (111), 40.5° (200), 70.1° (311) and 73.8° (222) were formed when the temperature rises from 140 °C to 200 °C. The observation above is well consistent with the H_2_-TPR characterization^38^. As a result, the assembled photo-thermal catalyst of Ru/MnO_x_ was composed of MnO phase and Mn_3_O_4_ phase at 200 °C. The phases of materials could remain stable at least 4 h at 200 °C, implying that the Ru/MnO_x_ photo-thermal catalyst composed of well-defined Ru/MnO/Mn_3_O_4_ can efficiently and stably catalyze CO_2_ hydrogenation to CH_4_ at reaction temperature. Therefore, the phase transformation of multiple-phase MnO_x_ is clearly indicative of Ru-mediated H-spillover effect. Furthermore, as shown in Supplementary Fig. 25, the catalytic activity of MnO_x_ supports surpasses that of the other specific manganese oxide alone. It indicates that the H-spillover effect in Ru/MnO_x_ can effectively transfer dissociated H to the support due to the multivalent states (Mn^2+^/Mn^3+^/Mn^4+^) with varied reducibility, thereby promoting the hydrogenation reaction.

In addition, to investigate the impact of photons on the H-spillover effect under photothermal conditions, we employed WO_3_ as a means to quantify the extent of H-spillover effect, by which the spillover hydrogen can migrate and readily react with yellow WO_3_, resulting in a dark coloration^39,40^. The experiment was conducted at 80 °C with a light intensity of 0.3 W cm^−2^ under 1 MPa H_2_ to ensure that the temperature induced by the photothermal effect remained below the designated temperature (Supplementary Fig. 26). As shown in Fig. 3f, it was revealed that the color of WO_3_ remained unchanged under both photothermal and thermal conditions. In contrast, the mixture of Ru/MnO_x_ and WO_3_ exhibited a darker color under photothermal conditions compared to thermal conditions. This observation suggests that under photothermal catalysis, the irradiation can enhance the H-spillover effect, thereby promoting the subsequent CO_2_ hydrogenation reaction.

Meanwhile, XPS was performed to gain insight into the composition and chemical valence. As can be observed in Fig. 3g, h and Supplementary Fig. 27, Ru 3*p* and Mn 2*p* peaks of Ru/MnO_*x*_ displayed a negative shift from 100 °C to 200 °C. Then, the Mn 2*p* and Ru 3*p* spectra remain unvaried within 4 h at 200 °C, which is in good agreement with the variable XRD measurements. Supplementary Fig. 28 shows that the binding energies of Ru shift to 462.7 eV, corresponding to Ru^0^ and the peaks of Mn^4+^ disappeared. The peak at 647 eV is the satellite peak of Mn^2+^. Such binding energy shifts indicate that Ru^3+^ and Mn^4+^ were reduced under the CO_2_/H_2_ mixed gas (H_2_:CO_2_ = 4:1), which is consistent with the H_2_-TPR characterization.

To better understand the reaction at molecular level and explore the impact of the involved photon on the reaction, CO_2_ hydrogenation was studied by FT-IR under different conditions (Fig. 3i and Supplementary Figs. 29, 30). For thermocatalysis, the typical peaks of monodentate carbonates (m-CO_3_^2−^, 1509 cm^−1^) and ν(C-H) vibration of CH_4_ (1305 cm^−1^) were apparently strengthened by increasing the reaction temperature^41,42^. Notably, the intermediate of formate species was observed at 1541 cm^−1^ (COOH*, *ν*(OCO)_as_) and 1373 cm^−1^ (COOH*, *ν*(OCO)_s_) when the reaction temperature increased up to 200 °C^43^. In contrast, upon light irradiation, the typical peaks of COOH* species appeared at a lower reaction temperature of 170 °C^44^. This finding further validated the synergy between photon energy and thermal energy on CO_2_ hydrogenation toward CH_4_, and COOH* is the most likely key intermediate during either thermo-catalysis or photo-thermal-catalysis. Hence, the involved photons were prone to accelerate the formation of intermediate species, thus can significantly reduce the activation energy of CO_2_ hydrogenation reaction and promote the formation of CH_4_.

**Fig. 4:**
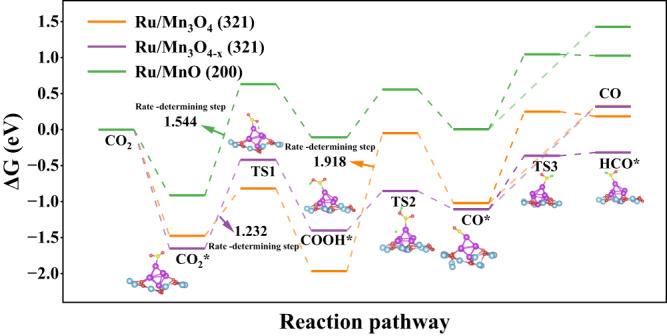
Gibbs free energy pathway for the formation of HCO* and CO from CO_2_ over Ru/Mn_3_O_4_ (321), Ru/Mn_3_O_4-x_ (321), and Ru/MnO (200). The blue, red, purple, yellow, and green spheres represent the Mn, O, Ru, C, and H atoms, respectively, in the calculation model.

To better understand the mechanism of the superior performance, first-principles density functional theory calculations were carried out on the basis of the models of Ru/Mn_3_O_4_ (321) slabs, Ru/MnO (200) slabs and Ru/Mn_3_O_4−x_ (321) slabs that simulated the partial reduction of Mn_3_O_4_ by the H-spillover effect (Supplementary Fig. 31)^45^. As shown in Fig. 4 and Supplementary Fig. 32–34, Ru/Mn_3_O_4−x_ has a more negative Gibbs free energy (Δ*G*) than both Ru/Mn_3_O_4_ and Ru/MnO during the adsorption of CO_2_, indicating a strong adsorption capacity for CO_2_, which is beneficial for CO_2_ hydrogenation (Δ*G* = −0.914 eV, Ru/MnO; Δ*G* = −1.475 eV, Ru/Mn_3_O_4_; Δ*G* = −1.651 eV, Ru/Mn_3_O_4−x_). Afterwards, notable variations for the subsequent CO_2_ hydrogenation were observed among Ru/MnO, Ru/Mn_3_O_4_, and Ru/Mn_3_O_4-x_. The formation of COOH* from CO_2_* is a rate determining step (RDS) for CO_2_ hydrogenation over Ru/Mn_3_O_4−x_ and Ru/MnO, which requires 1.232 and 1.544 eV, respectively. The protonation and subsequent dehydration of COOH* results in the generation of the intermediate of CO*, which is the RDS for the Ru/Mn_3_O_4_, (ΔG = 1.918 eV for Ru/Mn_3_O_4_). Notably, compared to HCO* formation, the CO* desorption from the catalytic surface as CO is relatively difficult for all the samples^46^. As a result, it is favorable to yield CH_4_ via further hydrogenation. It is worth mentioning that in the process of CO_2_ hydrogenation, Δ*G* of RDS over Ru/Mn_3_O_4−x_ (1.232 eV) is obviously lower than that on Ru/Mn_3_O_4_ (Δ*G* = 1.918 eV) and Ru/MnO (Δ*G* = 1.544 eV), thus facilitating the subsequent hydrogenation steps toward CH_4_. Moreover, as shown in Supplementary Fig. 35, compared with Ru/Mn_3_O_4−x_ (321) slabs that simulated dark state, the conduction band that simulated the light state moved to the low energy region, indicating that the involved photons were conducive to electron transfer, which is favorable to CO_2_ hydrogenation toward CH_4_^47–49^. Together with the FT-IR spectroscopic characterization above, it was rationalized that the synergy between photon energy and thermal energy favored the formation of COOH*, thus exerting a positive impact on the CO_2_ methanation over Ru/MnO_x_, which is generated by the partial reduction of Mn_3_O_4_ by Ru-mediated H-spillover effect in CO_2_ hydrogenation^50^.

Based on the spectroscopic and theoretical investigations above, a possible mechanism for photo-thermal-catalytic CO_2_ hydrogenation over Ru/MnO_x_ was proposed. The deposited Ru species is highly efficient for H_2_ dissociation. Benefitting from the strong interaction between Ru and MnO_x_, Ru-mediated H-spillover effect facilitated the structural evolution of Ru/MnO_x_ into well-defined Ru/MnO/Mn_3_O_4_ at low temperature, and the synergy between photon energy and thermal energy could facilitate further hydrogenation of the adsorbed CO_2_ molecules over Ru and MnO_x_ via accelerating the formation of the key intermediate COOH*. Critically, compared to the process of CO evolution from *CO, Ru/Mn_3_O_4−x_ is energetically favored for catalyzing *CO hydrogenation toward *HCO, thus promoting the subsequent hydrogenation toward the eventual product of CH_4_ with high activity and selectivity.

## Discussion

To summarize, a nanostructured Ru/MnO_x_ photo-thermal catalyst composed of well-defined Ru/MnO/Mn_3_O_4_ at reaction temperature was assembled for CO_2_ hydrogenation toward CH_4_. The catalyst illustrated a considerable CO_2_ conversion of 66.8% with a superior selectivity of 99.5% and a CH_4_ production rate of 166.7 mmol g^−1^ h^−1^ at 200 °C. A series of spectroscopic characterizations and theoretical investigations revealed that benefitting from the strong metal-support interaction between Ru and MnO_x_, Ru-mediated H-spillover facilitated the structural evolution of Ru/MnO_x_ into Ru/MnO/Mn_3_O_4_ at low temperature, and the synergy between photon energy and thermal energy promoted the yield of CH_4_ via reducing the activation energy of reaction and facilitating the formation of COOH* species. This work opens up a new way for photo-thermal-enhanced CO_2_ hydrogenation toward CH_4_.

## Methods

### Chemicals

MnSO_4_·H_2_O (99.99%), MnO (99.9%), MnO_2_ (99.95%), Mn_2_O_3_ (99.9%), WO_3_ (99.99%) and Ammonium acetate (99.99%) were purchased from Aladdin Chemical Reagent, Ltd. Acetylacetone (99.5%) was provided by Alfa Aesar Chemical Co. Ltd. H_2_SO_4_ (AR), CH_3_COOH (AR) and CH_3_OH (99.9%) was purchased from Sinopharm Chemical Reagent Co., Ltd. Ruodium (III) chloride hydrate (99%, Ru 37-40%) and NaOH (96%) were obtained from Beijing InnoChem Science & Technology Co., Ltd. Mn_3_O_4_ (99.95%) was obtained from Shanghai Macklin Biochemical Technology Co., Ltd. CO_2_ (99.995%) and H_2_(99.9995%) were provided by Air Liquid Houlding Co., Ltd., China. Deionized water was used in all the experiments.

### Materials synthesis

For the synthesis of MnO_x_, 1 mmol of MnSO_4_·H_2_O and 120 mmol of NaOH were dissolved in 30 mL of deionized water with stirring for 0.5 h. The suspension was subsequently transferred into a 100 mL Teflon liner autoclave. The autoclave was heated at 393 K for 12 h, and then cooled to room temperature. The precipitate was washed with distilled water several times until pH = 7, followed by drying in a vacuum at 333 K overnight to obtain the final product.

For the synthesis of Ru/MnO_x_, 0.1 g MnO_*x*_, 10 mL of CH_3_OH and suitable amount of RuCl_3_·3H_2_O (0.02, 0.04, 0.06, 0.08, 0.1, 0.12 mmol) were added in 50 mL of deionized water with stirring in a glass reactor (250 mL) with a quartz window. The chamber was evacuated and then filled with Ar of 1 atm and irradiated with 300 W UV–Xe lamp for 1 h in 3 W cm^−2^. The precipitate was washed with distilled water several times, followed by drying in a vacuum at 333 K overnight to obtain the final product. Unless otherwise specified, Ru/MnO_x_ indicates that the added amount of RuCl_3_·3H_2_O is 0.1 mmol.

Ru/MnO, Ru/Mn_2_O_3_, Ru/Mn_3_O_4_, and Ru/MnO_2_ were synthesized through an identical procedure. The major difference was that the corresponding commercial support was added to the reaction. 0.1 g MnO, Mn_2_O_3_, Mn_3_O_4_ or MnO_2_, 10 mL of CH_3_OH, and 0.1 mmol RuCl_3_·3H_2_O were added in 50 mL of deionized water with stirring in a glass reactor (250 mL) with a quartz window. The chamber was evacuated and then filled with Ar of 1 atm and irradiated with 300 W UV-Xe lamp for 1 h in 3 W cm^−2^. The precipitate was washed with distilled water several times, followed by drying in a vacuum at 333 K overnight to obtain the final product.

### Photo-thermal CO_2_ hydrogenation

Photo-thermal CO_2_ hydrogenation experiments were carried out in stainless steel reactor of 180 mL (CEL-MPR, Beijing China Education Au-Light Co., Ltd.). In a typical experiment, 15 mg of the catalyst powder was dispersed in 10 mL water in reactor, and the reactor was heated at 333 K overnight to volatilize the solvent and the thin film of catalyst was formed for further experiment (the thickness of the catalyst bed was 25.3 ± 4 µm). Prior to photo-thermal reaction, the reactor was sealed and the air in it was substituted with CO_2_ of 1 MPa three times and then CO_2_/H_2_ mixed gas of 1 MPa with desired ratio was charged at room temperature. Then, the external heating and the 300 W UV-Xe lamp (Beijing China Education Au-Light Co., Ltd) with an intensity of 2.5 W cm^−2^ both contributed to maintaining the reactor temperature at 200 °C. After the desired reaction time, the gas products were detected by a gas chromatograph (Agilent GC-8860) and calibrated with a standard gas mixture. To detect liquid products, 10 mL of water was injected into the system after the reaction. Possible liquid products such as methanol, ethanol, acetic acid, and acetaldehyde were detected with an Agilent Technology 7890B gas chromatography system with a flame ionization detector using a DB-WAX-UI column. The possible product formic acid was analyzed by HPLC (Waters 2695) equipped with Aminex HPX-87H column, UV/visible detector (WATER2489), and the mobile phase was 5 mM sulfuric acid and the flow rate of 0.7 mL/min. The amount of HCHO was analyzed by using the acetylacetone color-development method. Specifically, 1 mL of the as-prepared acetylacetone solution was mixed with 4 mL of the liquid product in a glass bottle, and heated for 5 min in boiling water. The yellow color of the mixed solution was then investigated. Afterwards, a specific amount of solution was taken out, and examined the UV–vis absorption spectrum by using a Shimadzu UV-2700 spectrophotometer. Through the absorbance intensity at 413 nm, the HCHO concentration was obtained. Typically, 100 mL of acetylacetone solution was first prepared by dissolving 15 g of ammonium acetate, 0.3 mL of acetic acid, and 0.2 mL of acetylacetone in water, and was stored in refrigerator with 2–6 °C.

The photothermal CO_2_ conversion is also performed in the fixed-bed reactor (CEL-GPPCM, Beijing China Education Au-Light Co., Ltd.) at 200 °C. 150 mg of catalyst and CO_2_/H_2_ mixed flow (20 mL min^−1^/80 mL min^−1^) were used. A 300 W UV-Xe lamp (Beijing China Education Au-Light Co., Ltd) was used as the light source for the reaction (light intensity: 2.5 W cm^-2^). The products in the effluent gas were periodically analyzed by using a gas chromatograph (GC-7920, Beijing China Education Au-Light Co., Ltd.). STY of CH_4_ (${{{\rm{mol}}}_{{CH}_{4}}}$ g^−1^ h^−1^), was calculated according to the following equation

1 $${{{{{{\rm{CH}}}}}}}_{4}{{{{{\rm{STY}}}}}}=\frac{{F}_{{CO}_{2},{in}}\times {X}_{{CO}_{2}}\times {S}_{{CH}_{4}}}{{W}_{{cat}}\times {V}_{m}}$$

where ${{F}_{{{\rm{CO}}}_{2}}}$, it is the volumetric flow rate of CO_2_, ${{X}_{{{\rm{CO}}}_{2}}}$ is the CO_2_ conversion, ${{S}_{{{\rm{CH}}}_{4}}}$ is the CH_4_ selectivity, *W*_cat_ is the overall mass of catalyst (g), and *V*_m_ is the ideal molar volume of CO_2_ at standard temperature and pressure.

### Materials characterization

The morphology of the samples was characterized by a Zeiss Sigma HD SEM and a JEOL JEM−2100F TEM. The high-angle annular dark-field scanning transmission electron microscope was operated by EM-ARM300F. A Rigaku Ultima VI XRD was employed to record the X-ray diffraction patterns with a scanning speed of 5°/min between 10° and 90°, which was operated at 25 kV and 35 mA with Cu Kα radiation. XPS measurements were operated on AXIS Supraelectron spectrometer with Al *K*α radiation. BET measurements were carried out by N_2_ at −196 °C in a Quadrasorb evo. Fourier transform-infrared spectroscopy was performed using Nicolet NEXUS670. UV–VIS–NIR diffuse reflectance spectra were obtained by a UV–VIS–NIR spectrophotometer (UV-3600 Plus, Shimadzu, Japan). Raman analysis was conducted on a Thermo Scientific DXR2 Smart Raman Spectrometer with a 532 nm laser. The adsorption isotherms of CO_2_ were determined at 273 K on a BELSORP-max II equipment. A liquid nitrogen-cooled charge-coupled device spectrometer (Princeton Instruments) and a microchannel plate photomultiplier tube (Hamamatsu) combined with time-correlated single photon counting technique (Edinburgh Instruments) were used for photon counting and lifetimes measurements under 375 nm excitation. The Ru contents were quantified by an inductively coupled plasma emission spectrometer (ICP-OES) on an Optima 8300. The H_2_-TPR were measured on Micromeritics AutoChem II chemisorption analyzer with a TCD detector, the sample was heated to 200 °C at 10 °C min^−1^ in an He flow (50 mL min^−1^) and then cooled to 80 °C. Next, the sample was heated to 700 °C at 20 °C min^−1^ in a 10% H_2_/He mixed flow (50 mL min^−1^) atmosphere and the outlet gas was detected by TCD. The CO_2_-TPD was measured on Micromeritics AutoChem II chemisorption analyzer with a TCD detector. The sample was heated to 200 °C at 10 °C min^−1^ in an He flow (50 mL min^−1^) and then cooled to 80 °C. Next, a 10% CO_2_/He mixed flow (50 mL min^−1^) was introduced to the catalyst bed for 0.5 h. The sample was then exposed to He (50 mL min^−1^) for 0.5 h to remove the weakly adsorbed CO_2_ from the surface. Finally, the sample was heated to 700 °C at 10 °C min^−1^ in a He atmosphere and the outlet gas was detected by TCD. The temperature of samples was recorded by an infrared thermal imaging camera (Fotrfic 315, Shanghai Thermal Imaging Technology Co., Ltd.). Considering the limited ability of the reactor window composed of aluminum oxide to penetrate 7–15 µm of infrared light, the image captures the internal temperature of the reactor by quickly removing the reactor window. Variable temperature XRD measurements were collected by an X-ray diffraction patterns (D8 Advance). The samples were heated in CO_2_/H_2_ mixed flow (10 mL min^−1^/40 mL min^−1^) from 25 °C to 200 °C. Data collection after maintaining the specified temperature for 5 min. XPS measurements under variable temperature were operated on Thermo EXCALAB 250Xi electron spectrometer with Al Kα radiation. The catalysts were held on the sample holder and activated with illumination in the pretreatment chamber under CO_2_/H_2_ mixed flow (10 mL min^−1^/40 mL min^−1^) from 25 °C to 200 °C. The sample was then introduced into the ultrahigh-vacuum chamber for XPS measurement at room temperature after maintaining the specified temperature for 5 min. FT-IR spectra under variable temperature were recorded with a NICOLET iS50 FTIR spectrometer (Thermo SCIENTIFIC, USA) equipped with a high-temperature reaction chamber and a mercury cadmium telluride (MCT) detector at a resolution of 4 cm^−1^ and 32 scans per spectrum. The background spectrum was scanned after mixture gas (CO_2_:H_2_ = 1:4) was introduced. TG-MS analyses were performed on a thermogravimetric analyser (NETZSCH STA449 F3-QMS403D) instrument under air. The catalysts were held on the sample holder and the reactor was sealed until the air in it was substituted with CO_2_/H_2_ mixed flow (10 mL min^−1^/40 mL min^−1^). After that the background spectrum was recorded. Upon reaching the desired temperature through simultaneous external heating and irradiation, the system was maintained for 5 min. Subsequently, the light source was removed for FT-IR measurement. The thickness of the catalyst bed in the batch reactor was measured by laser scanning confocal microscopy LEXT OLS5100.

### H-spillover effect detection by WO_3_

In a typical experiment, a mixture containing 1 g of WO_3_ and 0.015 g of catalyst was placed in a quartz glass culture dish. Then the quartz glass culture dish was placed in stainless steel reactor of 180 mL (CEL-MPR, Beijing China Education Au-Light Co., Ltd.). Prior to photo-thermal reaction, the reactor was sealed, and the air was replaced by H_2_ for three times, followed by filling with H_2_ (1 MPa). Then, the external heating and the 300 W UV-Xe lamp (Beijing China Education Au-Light Co., Ltd) with an intensity of 0.3 W cm^−2^ both contributed to maintain the reactor temperature at 80 °C. After the desired reaction time, the color change of the powder samples was recorded.

### The photoelectrochemical (PEC) tests

The photoelectrochemical tests of the samples were carried out on an electrochemical workstation (CHI660e, Chenhua Instrument, Shanghai, China) by using a three- electrode system. The catalyst was drop-coated on clean FTO glass, which was used as a working electrode, while Pt and Ag/AgCl electrodes acted as counter and reference electrodes, respectively. A 300 W Xe lamp (Aulight, Beijing) acted as the light source and all of the electrochemical tests were carried out in 0.1 mol L^−1^ sodium sulfate solution.

### Computational methods

We have employed the Vienna Ab initio Simulation Package to perform all density functional theory (DFT) calculations. The elemental core and valence electrons were represented by the projector augmented wave method and plane-wave basis functions with a cutoff energy of 400 eV. Generalized gradient approximation with the Perdew–Burke–Ernzerh of (GGA-PBE) exchange-correlation functional was employed in all the calculations. Geometry optimizations were performed with the force convergency smaller than 0.05 eV/Å. The spin-polarization effect was also considered. A climbing image nudged elastic band method was used to locate the transition states with the same convergence standard. The spin-polarization effect was also considered. The DFT-D3 empirical correction method was employed to describe van der Waals interactions. The DFT + U approach was introduced to treat the highly localized Mn 2*p* states, using parameters of *U*–*J* = 4. For FM of Mn_3_O_4_ and MnO, the initial magnetic moments of Mn atoms were set to be +5 μB. Monkhorst-Pack k-points of 1 × 1 × 1 and 2 × 2 × 1 were applied for all the surface calculations of Ru-Mn_3_O_4_ and Ru-MnO. Half atoms at bottom were fixed in all the calculation. The Gibbs free energy was calculated by the following equation: Δ*G* = Δ*E* + Δ*E*_ZPE _− *T*Δ*S,* where the value of Δ*E*, Δ*E*_ZPE_, and Δ*S* denotes the changes of DFT energy, the zero-point energy and the entropy at 473.15 K, respectively.

### Supplementary information

Supplementary Information

Peer Review File

### Source data

Source Data

## Data Availability

The data supporting the findings of this work are available within the article and its Supplementary Information files. All the data reported in this work are available from the authors. Source data are provided with this paper.
