# Supplementary material for: Photo-thermal coupling to enhance CO2 hydrogenation toward CH4 over Ru/MnO/Mn3O4
Source: Nat Commun. 2024 Feb 6;15:1109. doi: 10.1038/s41467-024-45389-7 (PMC10847166; doi:10.1038/s41467-024-45389-7)
Supplement: Supplementary file 1 — Supplementary Information [file 41467_2024_45389_MOESM1_ESM.pdf]

Supporting Information

Photo-thermal coupling to enhance CO_2 hydrogenation toward CH_4 over $Ru/MnO/Mn_3O_4$

Jianxin Zhai[1,2], Zhanghui Xia[1,2], Baowen Zhou[3]*, Haihong Wu[1,2]*, Teng Xue[1,2], Xiao Chen[1,2], Jiapeng Jiao[1,2], Shuaiqiang Jia[1,2], Mingyuan He[1,2]*, and Buxing Han[1,2,4]*

1. Shanghai Key Laboratory of Green Chemistry and Chemical Processes, State Key Laboratory of Petroleum Molecular & Process Engineering, School of Chemistry and Molecular Engineering, East China Normal University, Shanghai 200062, China
2. Institute of Eco-Chongming, Shanghai 202162, China
3. Key Laboratory for Power Machinery and Engineering of Ministry of Education, Research Center for Renewable Synthetic Fuel, School of Mechanical Engineering, Shanghai Jiao Tong University, Shanghai 200240, China
4. Beijing National Laboratory for Molecular Sciences, CAS Key Laboratory of Colloid and Interface and Thermodynamics, CAS Research/Education Center for Excellence in Molecular Sciences, Institute of Chemistry, Chinese Academy of Sciences, Beijing, 100190, China

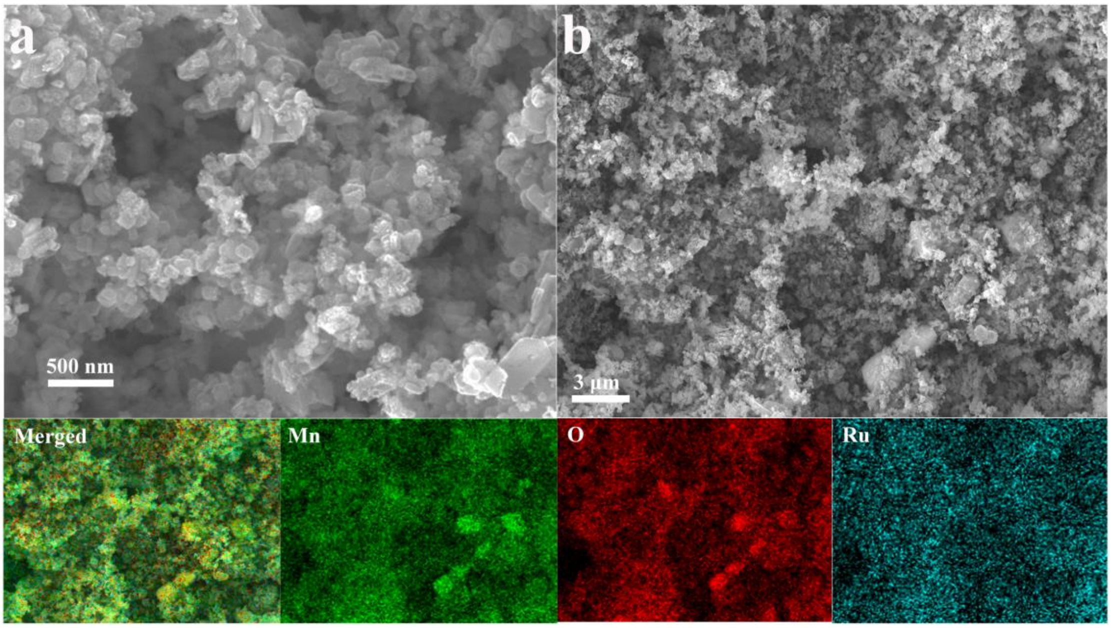

Supplementary Fig. 1 SEM image of (a) Ru/MnO_x; (b) The elemental mapping of Ru/MnO_x.

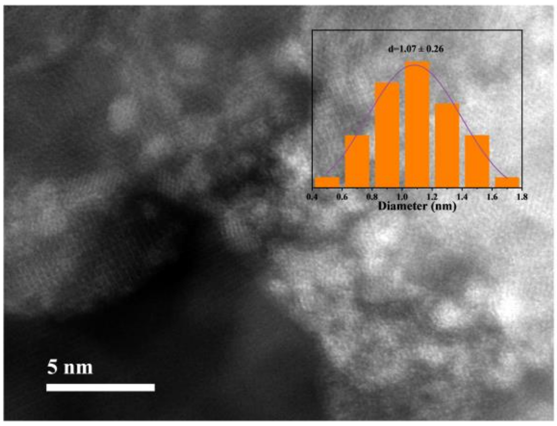

Supplementary Fig. 2 The high angle annular dark-field scanning transmission electron microscope (HAADF-STEM) image of the Ru/MnO_x catalyst.

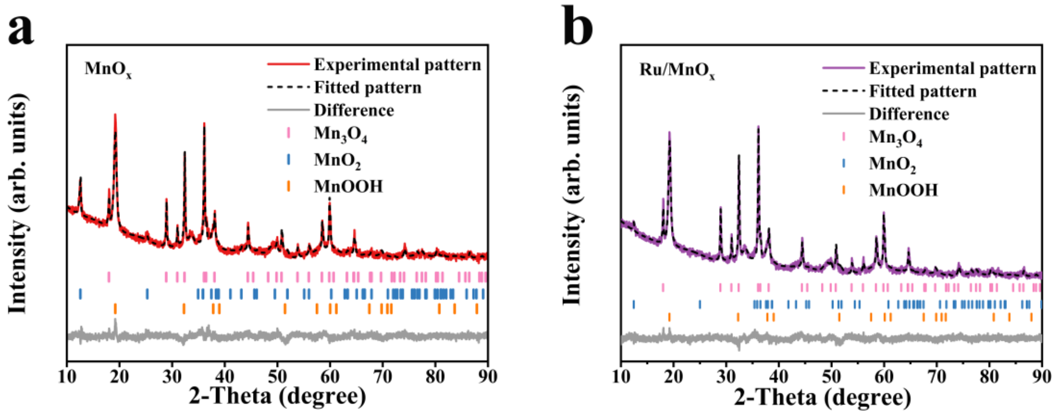

Supplementary Fig. 3 Rietveld refinement result of XRD patterns: (a) MnO_x; (b) Ru/MnO_x.

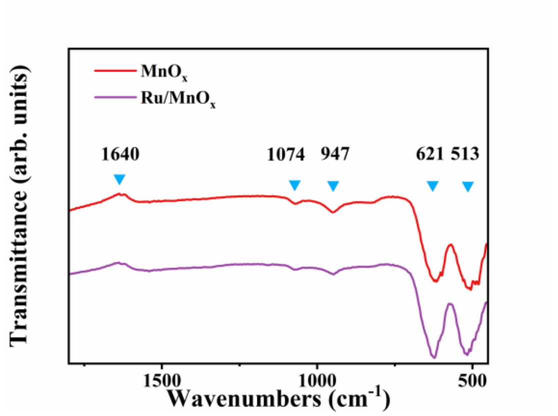

Supplementary Fig. 4 FT-IR spectra of MnO_x and Ru/MnO_x.

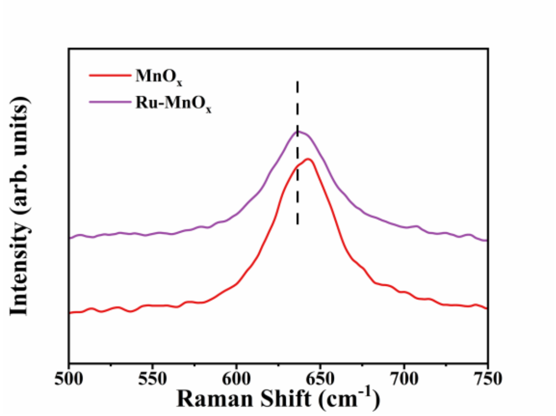

Supplementary Fig. 5 Raman spectra of MnO_x and Ru/MnO_x.

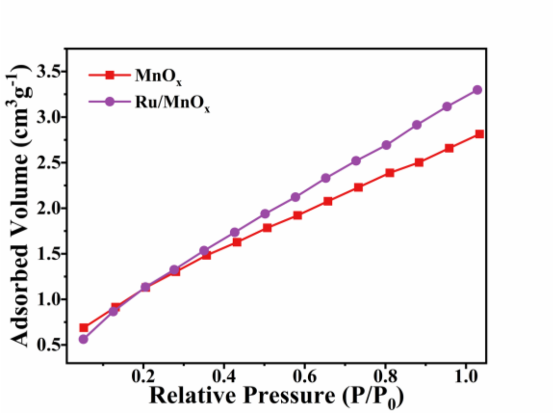

Supplementary Fig. 6 CO_2 adsorption isotherms of MnO_x and Ru/MnO_x.

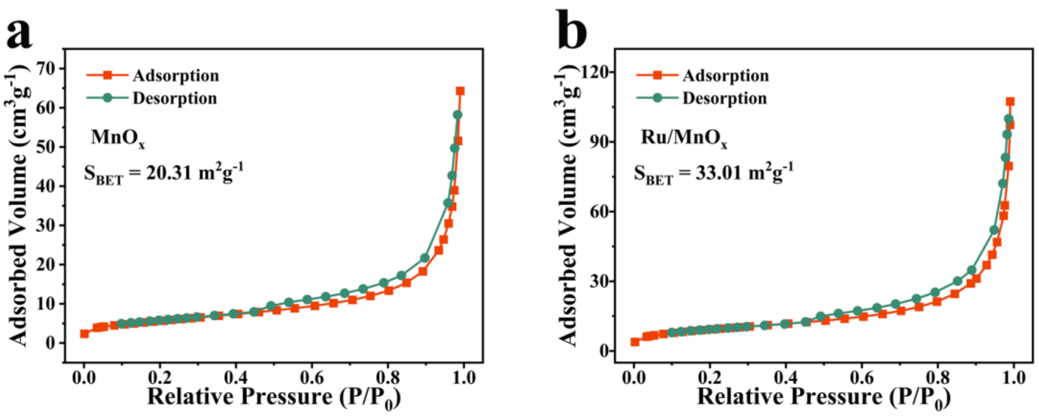

Supplementary Fig. 7 N_2 adsorption-desorption isotherms of (a) MnO_x; (b) Ru/MnO_x.

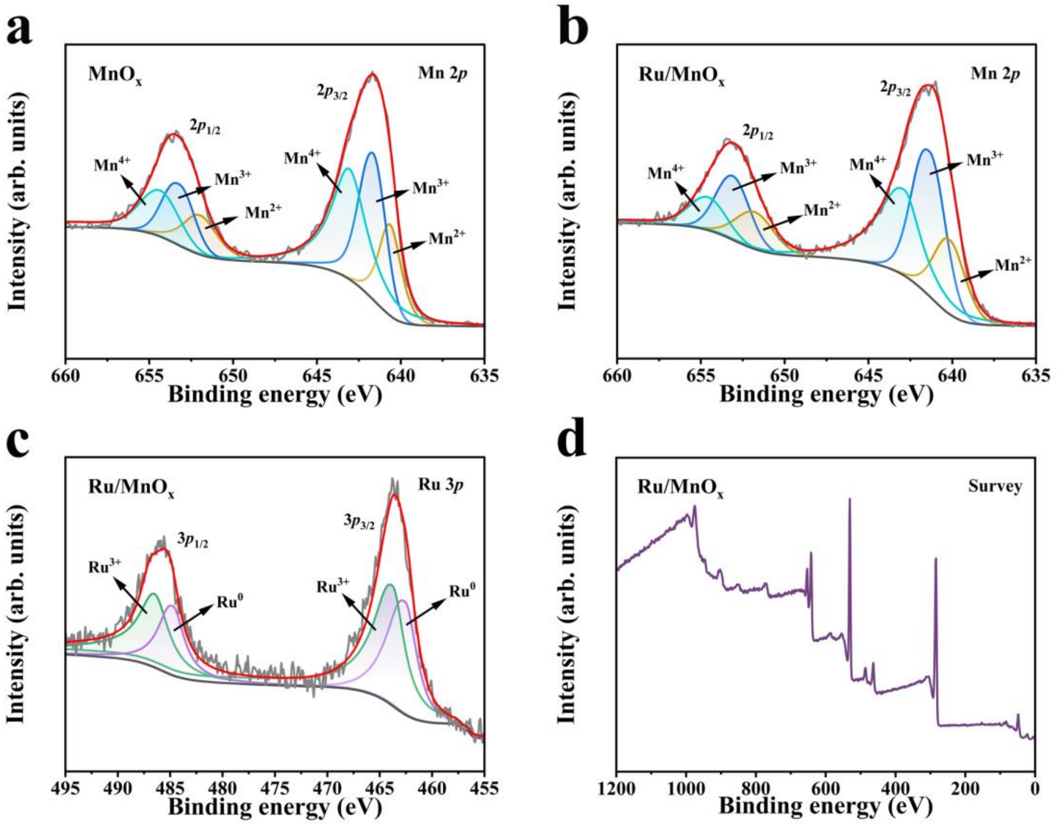

Supplementary Fig. 8 (a-b) High-resolution Mn 2*p* XPS spectra of MnO_x and Ru/MnO_x; (c) High-resolution Ru 3*p* XPS spectra of Ru/MnO_x; (d) XPS survey spectrum of Ru/MnO_x.

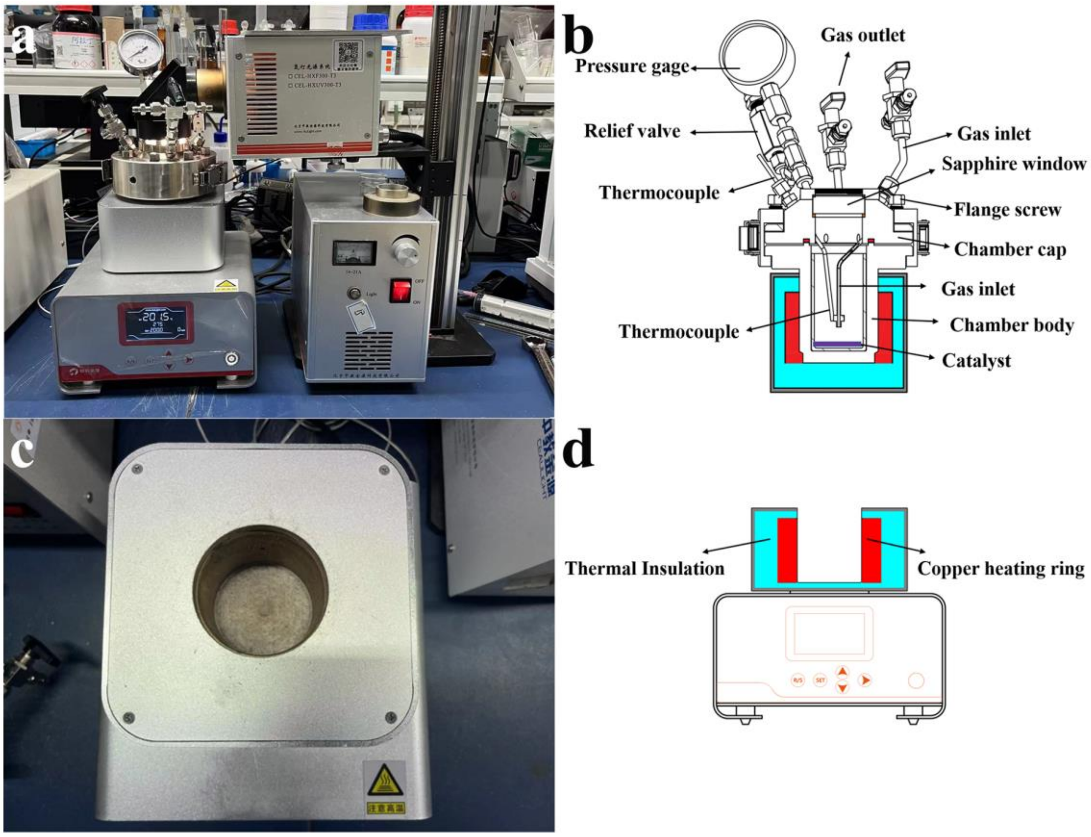

Supplementary Fig. 9 (a) Photograph of the apparatus setup for photo-thermal CO_2 experiments in the batch reactor; (b) Schematic illustration of the photo-thermal reactor; (c) and (d) Schematic illustration of the heating system.

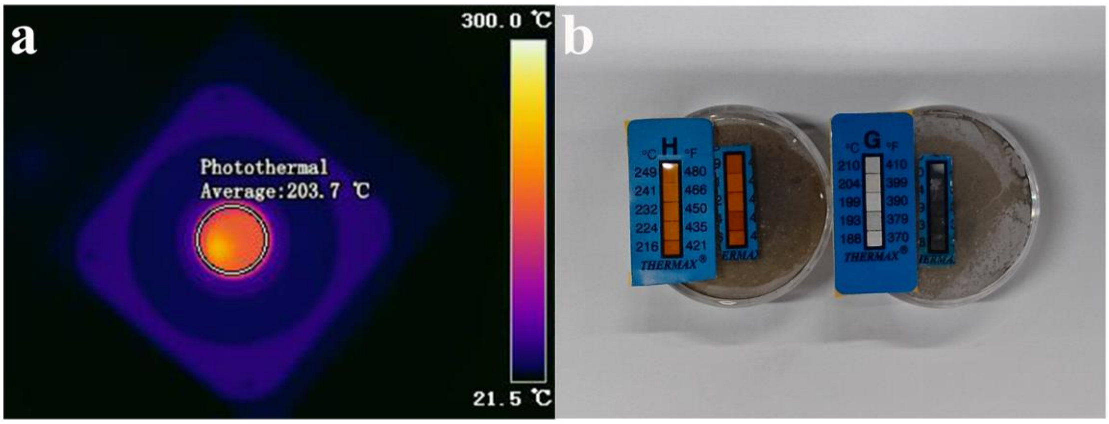

Supplementary Fig. 10 (a) Infrared thermal images captured for the catalyst surface temperature under 2.5 W cm^{-2} irradiation, 0.1 MPa and external heating (Set temperature: 200 °C); (b)The temperature at the bottom of the catalyst, measured using a commercially available thermochromic temperature indicator.

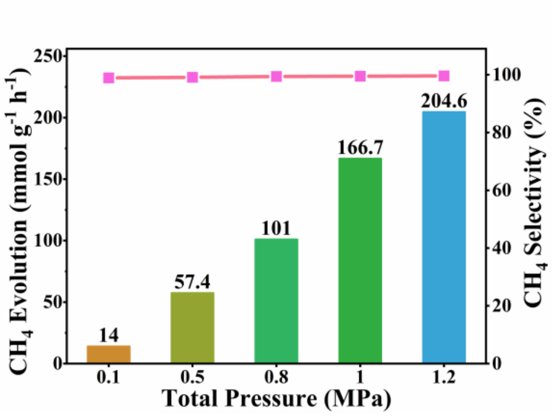

Supplementary Fig. 11 Influence of total pressure on CH_4 evolution rate over Ru/MnO_x; Reaction conditions: 15 mg of catalyst, full-arc 300 W UV-xenon lamp, 2.5 W cm^{-2}, 200 °C, irradiation time 4 hours, H_2/CO_2 =4/1.

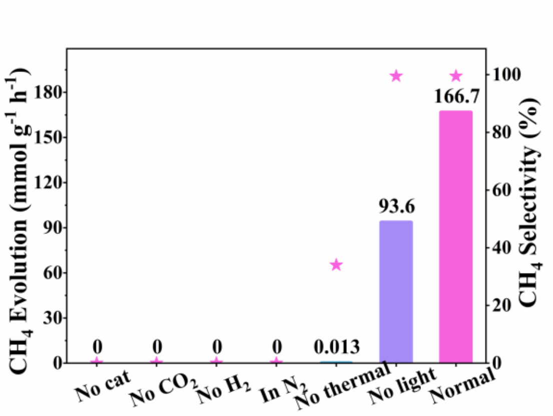

Supplementary Fig. 12 Control experiments for Ru/MnO$_x$ under various conditions. Reaction conditions: 15 mg of catalyst, full-arc 300 W UV-xenon lamp, 2.5 W cm^{-2}, 200 ℃, irradiation time 4 hours, initial pressure 1 MPa (H_2/CO_2 =4/1).

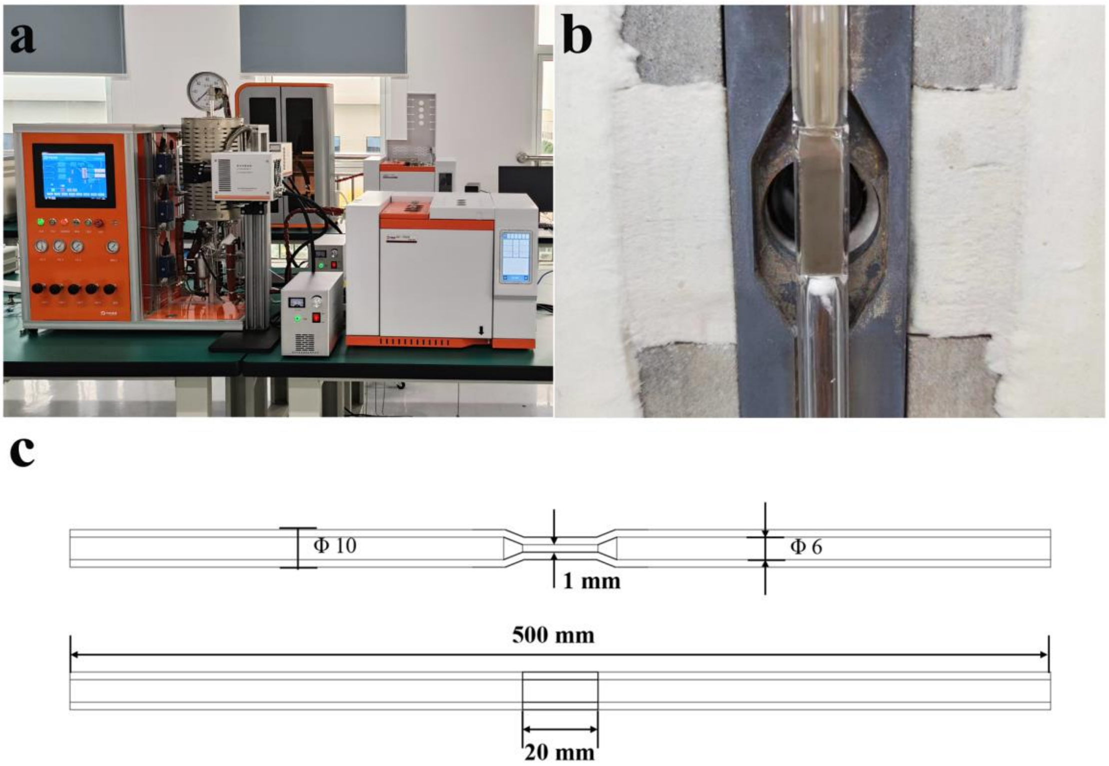

Supplementary Fig. 13 The images of (a) the photo-thermal catalytic performance evaluation process carried out in the flow reaction system and (b) the fixed-bed quartz tube reactor. (c) Dimensions of the fixed-bed quartz tube reactor.

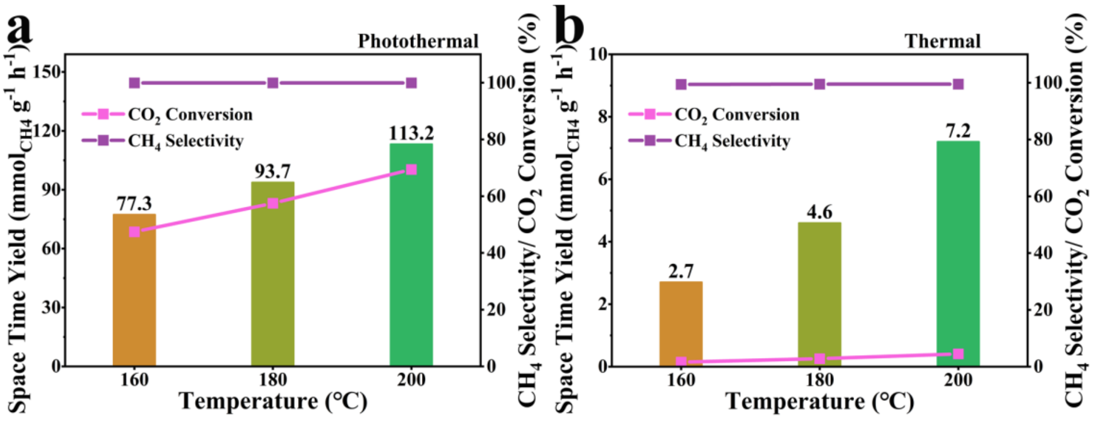

Supplementary Fig. 14 Temperature-dependent space time yield of CH_4 over Ru/MnO_x under photothermal (a) and thermal (b) conditions. Reaction conditions: 150 mg of catalyst, full-arc 300 W UV-xenon lamp, 2.5 W cm^{-2}, initial pressure 0.1 MPa, CO_2/H_2 mixture flow (10 mL min^{-1}/40 mL min^{-1}).

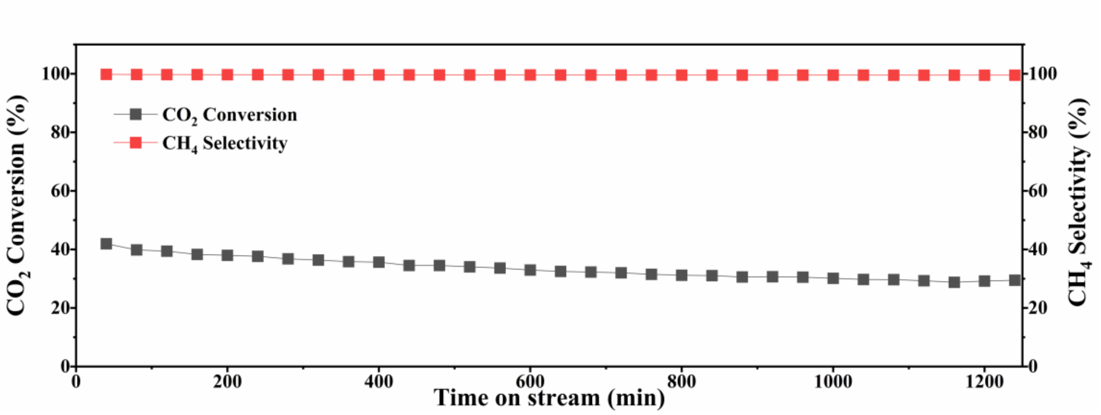

Supplementary Fig. 15 The photothermal catalytic performance of Ru/MnO_x catalyst in a fixed-bed reactor. Reaction conditions: 150 mg of catalyst, full-arc 300 W UV-xenon lamp, 2.5 W cm^{-2}, 200 °C, initial pressure 0.1 MPa, CO_2/H_2 mixture flow (20 mL min^{-1}/80 mL min^{-1}).

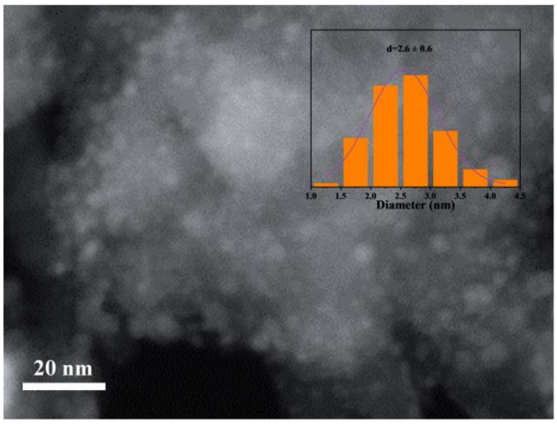

Supplementary Fig. 16 TEM image of Ru/MnO_x after reaction of 20 h at 200 °C under photothermal condition in the fixed-bed reactor.

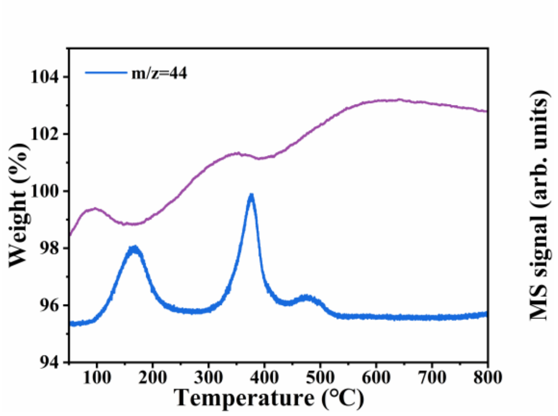

Supplementary Fig. 17 TG-MS analysis of Ru/MnO_x after reaction of 20 h at 200 °C under photothermal condition in the fixed-bed reactor.

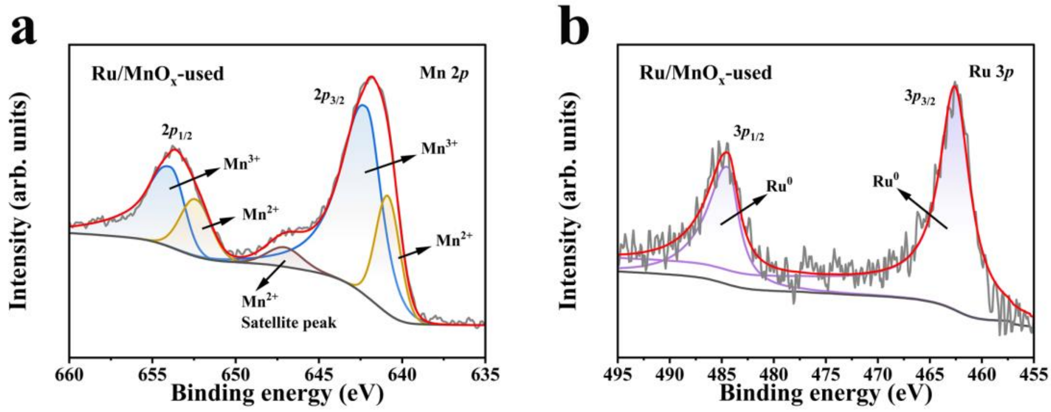

Supplementary Fig. 18 XPS spectra of Ru/MnO_x after reaction in 4 h at 200 ℃ in the batch reactor: (a) High-resolution of Mn 2*p* XPS spectra; (b) High-resolution of Ru 3*p* XPS spectra.

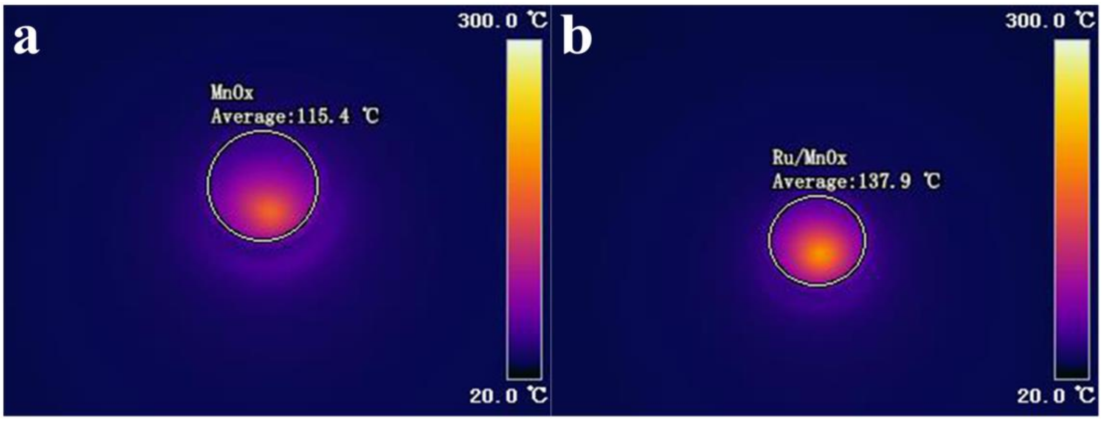

Supplementary Fig. 19 Infrared thermal images captured for (a) MnO_x and (b) Ru/MnO_x under 2.5 W cm^{-2} illumination.

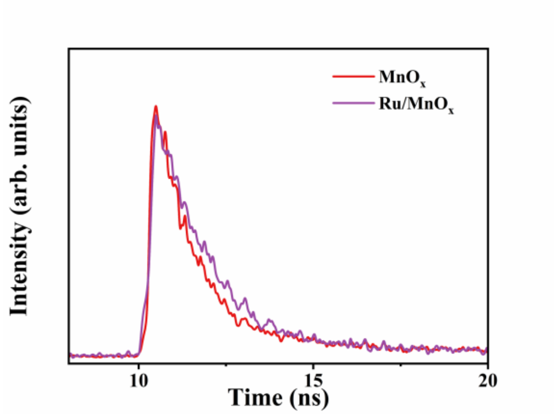

Supplementary Fig. 20 TRPL spectra of MnO_x and Ru/MnO_x.

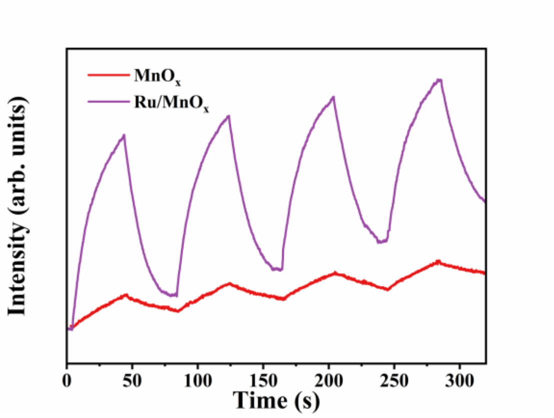

Supplementary Fig. 21 The periodic on/off photocurrent response spectra of MnO_x and Ru/MnO_x.

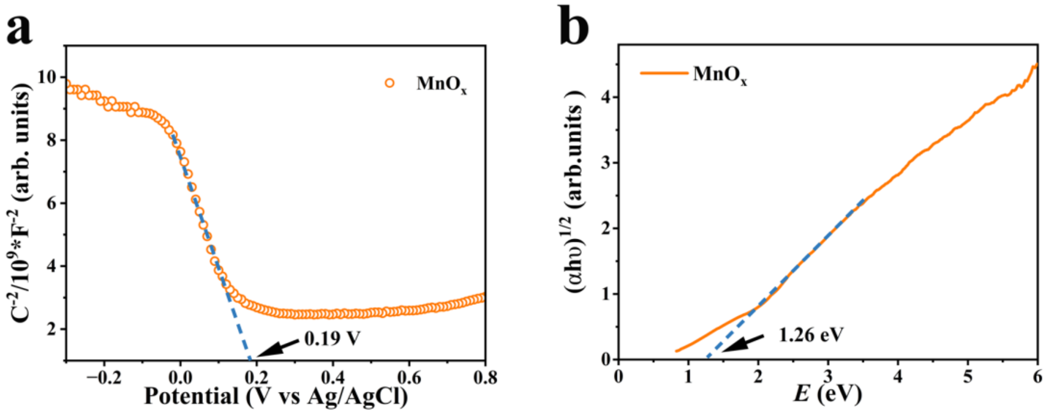

Supplementary Fig. 22 (a) Mott–Schottky plots of the MnO_x; (b) The bandgap value of the MnO_x.

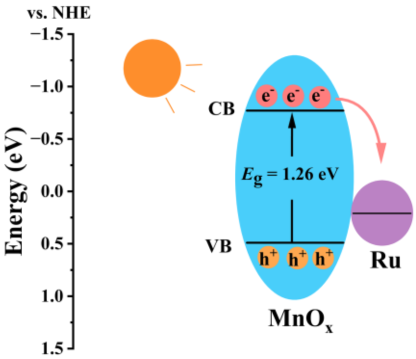

Supplementary Fig. 23 The work function of Ru and band structures of MnO_x.

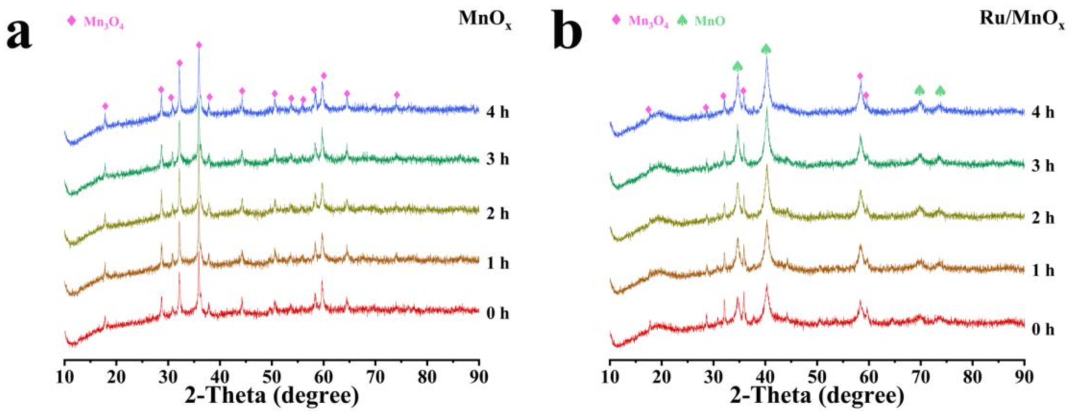

Supplementary Fig. 24 The different time of variable temperature XRD results in 20% CO_2/H_2 atmosphere at 200 °C: (a) MnO_x; (b) Ru/MnO_x.

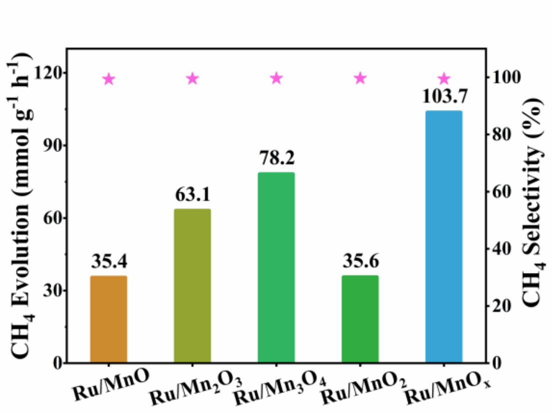

Supplementary Fig. 25 Influence of various manganese oxide on CH_4 evolution rate. Reaction conditions: 15 mg of catalyst, full-arc 300 W UV-xenon lamp, 2.5 W cm^{-2}, 200 °C, irradiation time 4 hours, initial pressure 1 MPa (H_2/CO_2 =1/1).

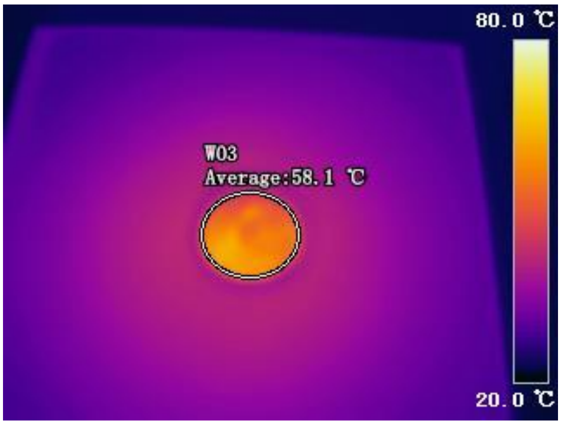

Supplementary Fig. 26 Infrared thermal images captured for WO_3 under 0.3 W cm^{-2} illumination.

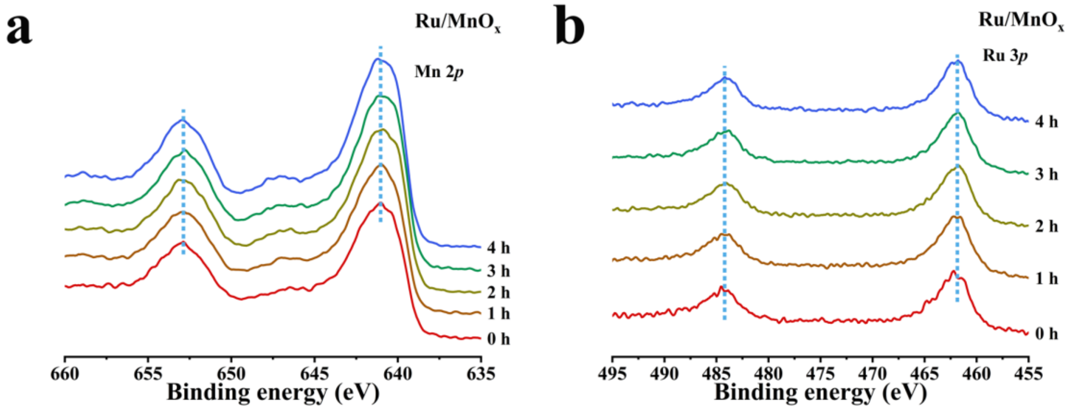

Supplementary Fig. 27 XPS spectra of Ru/MnO_x in 20% CO_2/H_2 atmosphere under variable time at 200 °C: (a) High-resolution of Mn 2*p* XPS spectra; (b) High-resolution of Ru 3*p* XPS spectra.

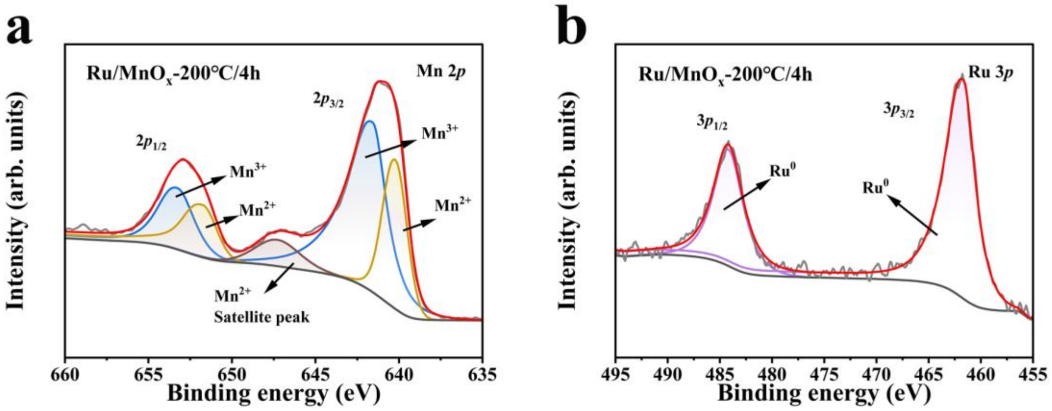

Supplementary Fig. 28 XPS spectra of Ru/MnO_x after reacting at 200 °C for 4 h in a 20% CO_2/H_2 atmosphere: (a) High-resolution of Mn 2*p* XPS spectra; (b) High-resolution of Ru 3*p* XPS spectra.

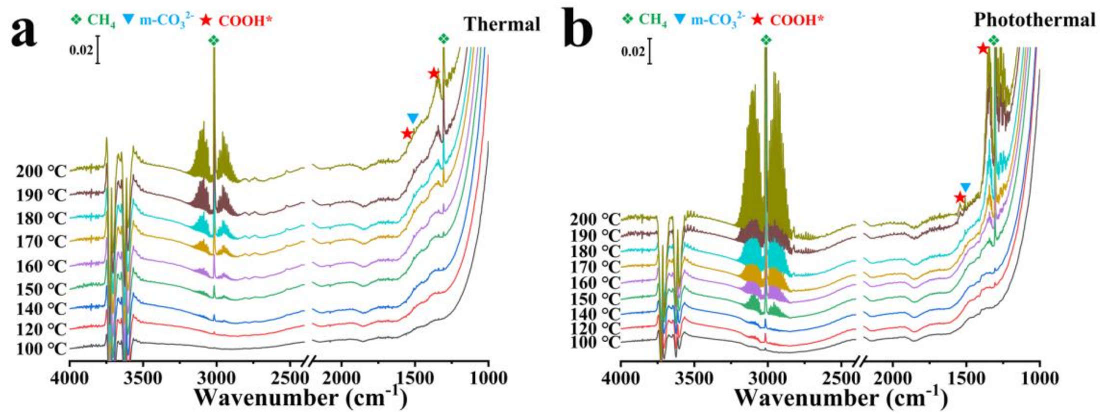

Supplementary Fig. 29 Spectra of FT-IR study of Ru/MnO_x at different conditions: (a) Effect of different temperature under thermal condition; (b) Effect of different temperature under photothermal conditions.

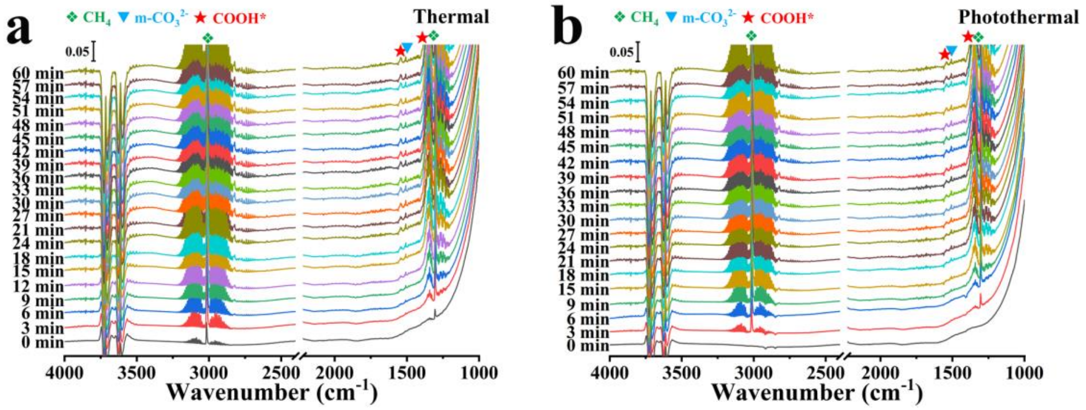

Supplementary Fig. 30 Spectra of FT-IR study of Ru/MnO_x at different conditions: (a) Effect of different time at 200 ℃ under thermal condition; (b) Effect of different time at 200 ℃ under photothermal conditions.

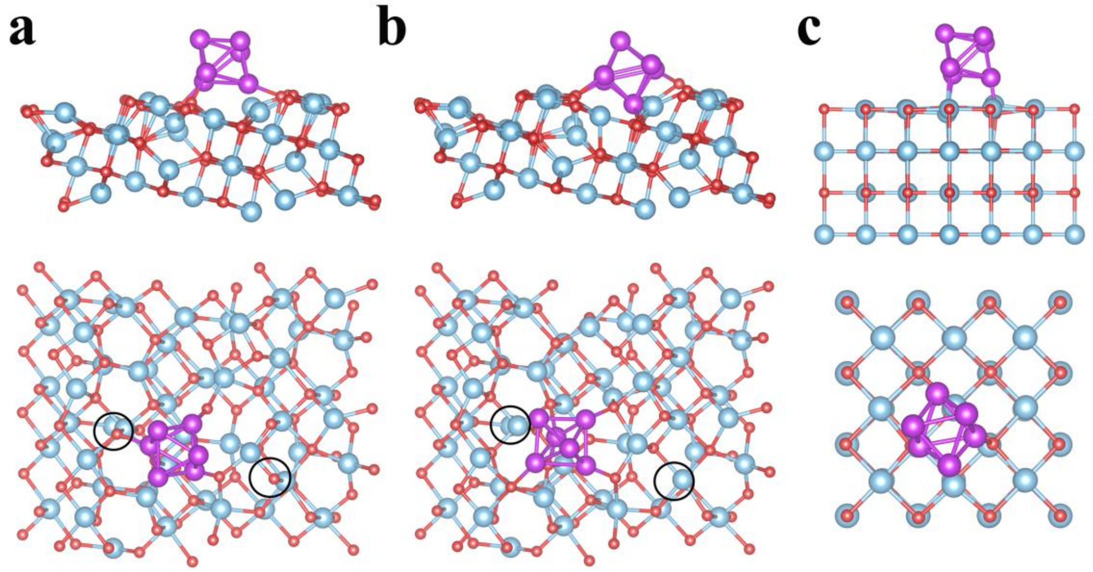

Supplementary Fig. 31 Calculation model of (a) Ru/Mn_3O_4 (321), (b) Ru/Mn_3O_{4-x} (321) and (c) Ru/MnO (200).

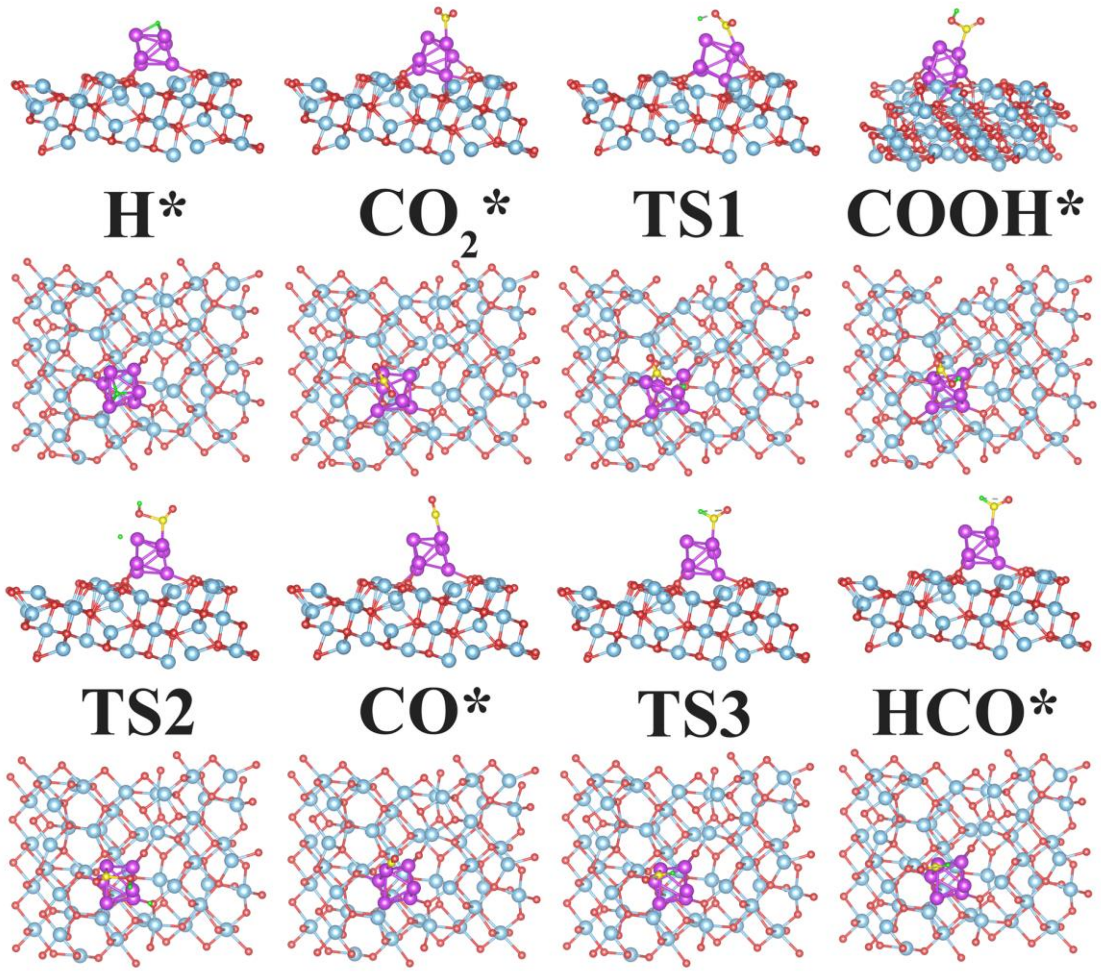

Supplementary Fig. 32 Adsorption configurations of all the involved species on Ru/Mn_3O_4 (321). The blue, red, purple, yellow, and green spheres represent the Mn, O, Ru, C, and H atoms, respectively.

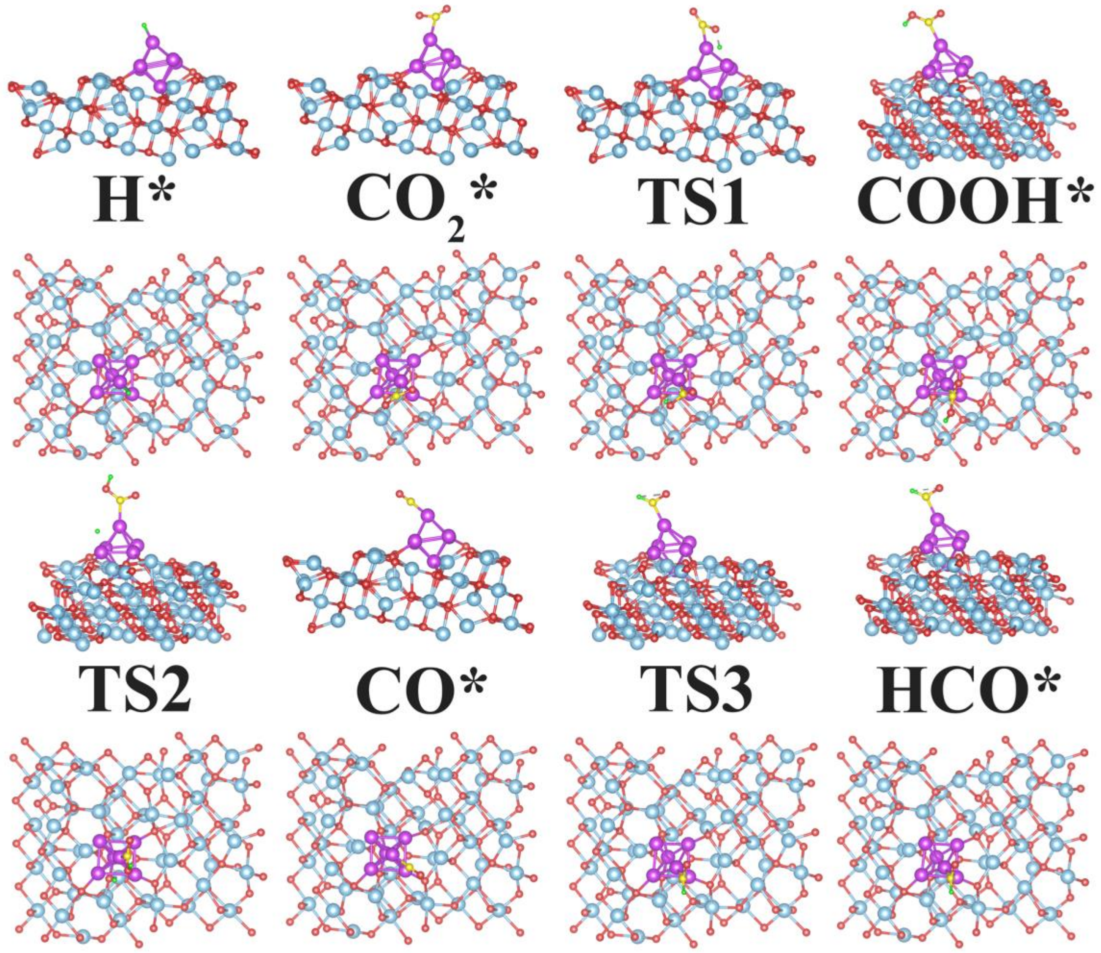

Supplementary Fig. 33 Adsorption configurations of all the involved species on Ru/Mn_3O_{4-x} (321). The blue, red, purple, yellow, and green spheres represent the Mn, O, Ru, C, and H atoms, respectively.

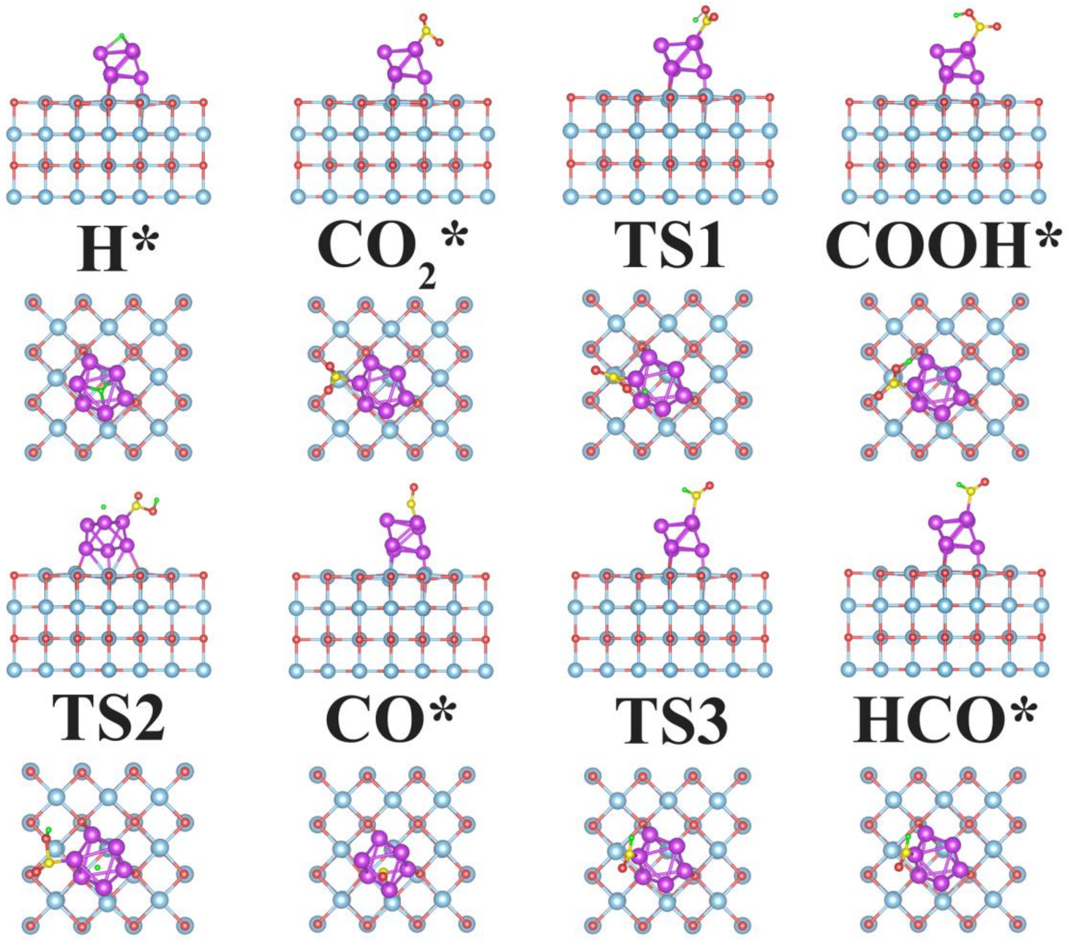

Supplementary Fig. 34 Adsorption configurations of all the involved species on Ru/MnO (200). The blue, red, purple, yellow, and green spheres represent the Mn, O, Ru, C, and H atoms, respectively.

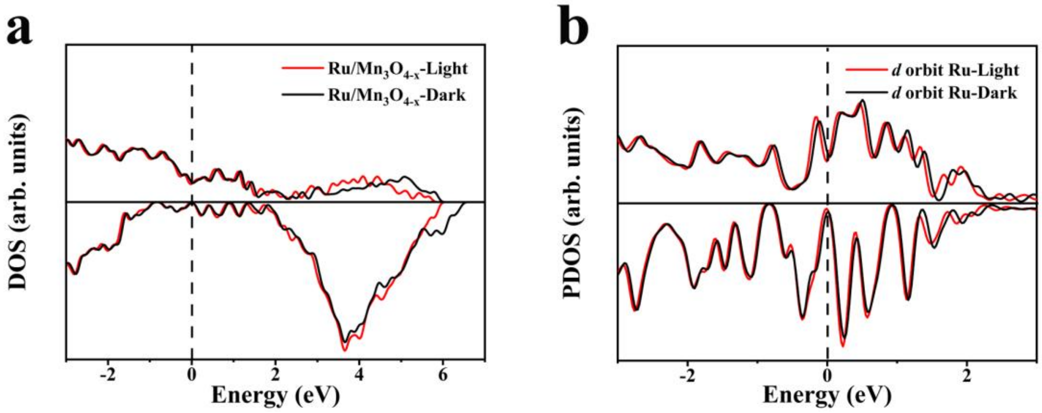

Supplementary Fig. 35 The calculated (a) densities of states and (b) projected densities of states for Ru/Mn_3O_{4-x} under dark and light conditions. Fermi levels are at 0 eV.

Supplementary Table 1 ICP-OES analysis of Ru/MnO_x.

Catalyst	Ru (wt%)	$RuCl_3 \cdot 3H_2O$ addition (mmol)
$1.4\%Ru/MnO_x$	1.4	0.02
$2.8\%Ru/MnO_x$	2.8	0.04
$4.0\%Ru/MnO_x$	4.0	0.06
$5.8\%Ru/MnO_x$	5.8	0.08
$7.3\%Ru/MnO_x$	7.3	0.1
$8.1\%Ru/MnO_x$	8.1	0.12

Supplementary Table 2 Crystal parameters and reliability factors of the refinement for MnO_x and Ru/MnO_x.

Sample	MnO_x			Ru/MnO_x		
Phase	Mn_3O_4	MnOOH	MnO_2	Mn_3O_4	MnOOH	MnO_2
Abundance (%)	69.023	25.976	5.002	72.528	27.544	0.928
Space group	I41/amd	P-3m1	C12/m1	I41/amd	P-3m1	C12/m1
a (Å)	5.7702(4)	3.2031(16)	5.1657(61)	5.7698(2)	3.2016(17)	5.1657(61)
b(Å)	5.7702(4)	3.2031(16)	2.8645(61)	5.7698(2)	3.2016(17)	2.8645(33)
c(Å)	9.4544(9)	4.6199(9)	7.0860(32)	9.4490(5)	4.6141(7)	7.0860(32)
Volume(Å^3)	314.796(62)	41.050(42)	104.17(18)	314.569(31)	40.959(44)	104.17(18)
R_{wp}		1.62%			1.71%	
R_p		1.25%			1.33%	
GOF		1.34			1.33	

Supplementary Table 3 The summarized CH_4 yields for recently reported photo-thermo-catalysts.

Catalysts	Metal loading (wt%)	H_2:CO_2 ratio	Pressure (Mpa)	Light sources	Light intensity (W cm^{-2})	Temperature (°C)	CH_4 production rate (mmol g^{-1} h^{-1})	CO_2 conversion (%)	CH_4 selectivity (%)	TOF (h^{-1})	Ref
Ru/MnO_x	7.3	4:1	1	300 W Xe lamp 200-1100 nm	2.5	200 (external heater)	166.7	66.8	99.5	232	This work
$Co_7Cu_1Mn_1O_x$(200)	—	3:1	0.1	300 W Xe lamp 300-1100 nm	0.234	200 (external heater)	14.5	27.45	85.3	—	1
Ru/Al_2O_3	2.4	4:1	0.08	1000 W Xe lamp	—	396	115	0.95	99.2	484	2
Cu_2O/Graphene		4:1	0.13	300 W Xe lamp	0.2	250 (external heater)	14.93 (Cu)	2.84	99	0.256	3
$Ru@Ni_2V_2O_7$	0.35	4:1	0.067	300 W Xe lamp	2	350	114.9	96.3	99.3	3340	4
$Ru/Mg(OH)_2$	11.5	1:1	0.1	300 W Xe lamp	1.8	—	44.85	1.68	69.5	56.7	5
Rh/Al	5	3:1	1.5	300 W Xe lamp	11.3	200 (external heater)	550	—	99	1132	6
21%Co/Al_2O_3	0.21	4:1	0.1	300 W Xe lamp 200-1100 nm	1.3	292	6.04	—	97.7	1.74	7
$Ru-TiO_x$	1.77	4:1	—	300 W Xe lamp	2	276	22.35	—	99.99	12.76	8
Ir@UiO66	0.14	4:1	0.1	300 W Xe lamp	2.3	250 (external heater)	19.9 (Flow reactor)	9.3	95	2876	9
8 % Ru/SiO_2	0.8	6:1	—	300 W Xe lamp	0.063	300 (external heater)	55.44 (Flow reactor)	51.8	99	70	10
$Ru-Al_2O_{3-x}$-L	0.7	4:1	0.1	300 W Xe lamp	2.27	236	0.84	86.47	99	1248	11
Ru/H_xMoO_{3-y}	4	1:1	—	300 W Xe lamp Vis-IR	0.75	140 (external heater)	20.8 (Flow reactor)	—	99	52.6	12

Supplementary References

1. He Z, *et al.* Photothermal CO_2 hydrogenation to hydrocarbons over trimetallic Co–Cu–Mn catalysts. *Green Chemistry* **23**, 5775-5785 (2021).
2. Grote R, *et al.* Collective photothermal effect of Al_2O_3-supported spheroidal plasmonic Ru nanoparticle catalysts in the sunlight-powered Sabatier reaction. *ChemCatChem* **12**, 5618-5622 (2020).
3. Mateo D, Albero J, García H. Photoassisted methanation using Cu_2O nanoparticles supported on graphene as a photocatalyst. *Energy & Environmental Science* **10**, 2392-2400 (2017).
4. Chen Y, *et al.* Cooperative catalysis coupling photo-/photothermal effect to drive Sabatier reaction with unprecedented conversion and selectivity. *Joule* **5**, 3235-3251 (2021).
5. Kong N, *et al.* Ruthenium nanoparticles supported on $Mg(OH)_2$ microflowers as catalysts for photothermal carbon dioxide hydrogenation. *ACS Applied Nano Materials* **3**, 3028-3033 (2020).
6. Fu G, *et al.* Rh/Al nanoantenna photothermal catalyst for wide-spectrum solar-driven CO_2 methanation with nearly 100% selectivity. *Nano Letters* **21**, 8824-8830 (2021).
7. Chen X, *et al.* MOF-templated preparation of highly dispersed Co/Al_2O_3 composite as the photothermal catalyst with high solar-to-fuel efficiency for CO_2 methanation. *ACS Applied Materials & Interfaces* **12**, 39304-39317 (2020).
8. Dong T, *et al.* Ru decorated TiO_x nanoparticles via laser bombardment for photothermal co-catalytic CO_2 hydrogenation to methane with high selectivity. *Applied Catalysis B: Environmental* **326**, 122176 (2023).
9. Tang Y, *et al.* Encapsulating Ir nanoparticles into UiO-66 for photo-thermal catalytic CO_2 methanation under ambient pressure. *Journal of Materials Chemistry A* **10**, 12157-12167 (2022).
10. Kim C, *et al.* Energy-efficient CO_2 hydrogenation with fast response using photoexcitation of CO_2 adsorbed on metal catalysts. *Nature Communications* **9**, 3027 (2018).
11. Liu X, *et al.* Strong interaction over Ru/defects-rich aluminium oxide boosts photothermal CO_2 methanation via microchannel llow-type system. *Advanced Energy Materials* **12**, 2201009 (2022).
12. Ge H, Kuwahara Y, Kusu K, Bian Z, Yamashita H. Ru/H_xMoO_{3-y} with plasmonic effect for boosting photothermal catalytic CO_2 methanation. *Applied Catalysis B: Environmental* **317**, 121734 (2022).
